# Silver Nanoparticle Protein Corona Composition in Cell Culture Media

**DOI:** 10.1371/journal.pone.0074001

**Published:** 2013-09-09

**Authors:** Jonathan H. Shannahan, Xianyin Lai, Pu Chun Ke, Ramakrishna Podila, Jared M. Brown, Frank A. Witzmann

**Affiliations:** 1 Department of Pharmaceutical Sciences, Skaggs School of Pharmacy and Pharmaceutical Sciences, University of Colorado Anschutz Medical Campus, Aurora, Colorado, United States of America; 2 Department of Cellular and Integrative Physiology, Indiana University School of Medicine, Indianapolis, Indiana, United States of America; 3 Department of Physics and Astronomy, Clemson University, Clemson, South Carolina, United States of America; Northwestern University Feinberg School of Medicine, United States of America

## Abstract

The potential applications of nanomaterials as drug delivery systems and in other products continue to expand. Upon introduction into physiological environments and driven by energetics, nanomaterials readily associate proteins forming a protein corona (PC) on their surface. This PC influences the nanomaterial’s surface characteristics and may impact their interaction with cells. To determine the biological impact of nanomaterial exposure as well as nanotherapeutic applications, it is necessary to understand PC formation. Utilizing a label-free mass spectrometry-based proteomics approach, we examined the composition of the PC for a set of four silver nanoparticles (AgNPs) including citrate-stabilized and polyvinlypyrrolidone-stabilized (PVP) colloidal silver (20 or 110 nm diameter). To simulate cell culture conditions, AgNPs were incubated for 1 h in Dulbecco’s Modified Eagle Medium supplemented with 10% fetal bovine serum, washed, coronal proteins solubilized, and proteins identified and quantified by label-free LC-MS/MS. To determine which attributes influence PC formation, the AgNPs were characterized in both water and cell culture media with 10% FBS. All AgNPs associated a common subset of 11 proteins including albumin, apolipoproteins, keratins, and other serum proteins. 110 nm citrate- and PVP-stabilized AgNPs were found to bind the greatest number of proteins (79 and 85 respectively) compared to 20 nm citrate- and PVP-stabilized AgNPs (45 and 48 respectively), suggesting a difference in PC formation based on surface curvature. While no relationships were found for other protein parameters (isoelectric point or aliphatic index), the PC on 20 nm AgNPs (PVP and citrate) consisted of more hydrophobic proteins compared to 110 nm AgNPs implying that this class of proteins are more receptive to curvature-induced folding and crowding in exchange for an increased hydration in the aqueous environment. These observations demonstrate the significance of electrostatic and hydrophobic interactions in the formation of the PC which may have broad biological and toxicological implications.

## Introduction

In biological environments, it has been recently established that nanomaterials interact readily with proteins, peptides, and lipids thus forming a protein corona (PC) on their surface [1], [2], [3]. The proteins which associate with the nanomaterial to form the PC are dependent on a number of variables including the biological media and the physicochemical characteristics of the nanomaterial [4], [5], [6]. The addition of these proteins on the surface of the nanomaterial can alter its bio-distribution, clearance, activity, and toxicity through modifications to its hydrodynamic size, shape, charge, and interfacial characteristics. The PC, rather than the nanoparticle solely, therefore may be responsible for the biological effects induced by a nanomaterial and could be utilized for the targeting of nanotherapeutics and the reduction of unintended toxicity [3], [7], [8]. Specifically, research has demonstrated that the PC may influence nanomaterial uptake by cells and distribution, alter cytotoxicity, and increase inflammation and oxidative stress [9], [10], [11], [12], [13], [14], [15]. Furthermore, in attempts to target nanotherapeutics the surface of nanomaterials have been functionalized with proteins, such as transferrin; however, upon introduction into a biological fluids, the targeting is lost due to the addition of the PC [16]. Therefore, an understanding of the PC is necessary to successfully utilize nanomaterials, implement nanotherapeutics, and limit off-target toxicity.

Silver nanoparticles (AgNPs) have been incorporated as antibacterial/antifungal agents in medical devices, textile engineering, water treatment, and have been integrated into the surfaces of many household appliances and food storage containers [17], [18]. The increased utilization of these particles however has raised concern regarding the human health effects following exposure. These AgNPs are colloidal in shape and exhibit their antibacterial/antifungal activity through the release of silver ions resulting in cytotoxicity. *In vitro* toxicity studies have demonstrated that exposure to AgNPs can result in mitochondrial damage, reactive oxygen species production, cell-cycle arrest, apoptosis, and cytotoxicity [19], [20], [21]. Animal studies have shown that exposure to AgNP induces inflammatory gene expression, cytokine production, cytotoxicity, and altered antioxidant status [22]. A variety of AgNPs are currently being produced comprising an array of sizes and surface coatings. Two surface coatings that are typically used are polyvinylpyrrolidone (PVP) and citrate. These surface coatings allow for steric or electrostatic stabilization of the AgNPs within solution and decrease aggregration of the nanoparticles. Differences in the size of AgNPs have been shown to be related to uptake and toxicity [23], [24], [25], whereas differences in coatings have demonstrated alterations in bacterial cytotoxicity related to surface charge [26]. Currently however it is unclear how differences in size and surface coatings may affect the formation and identity of the PC as well as its effect on nanotherapeutic applications and toxicity.

Screening the biocompatibility of nanoparticles typically utilizes a variety of *in vitro* techniques, which have often yielded inconsistent results. It is generally accepted that physicochemical characterization of the nanomaterial is needed to understand and predict toxicity, yet less attention has been given to the disparities between cell culture media and the subsequent effect on PC formation in the interpretation of *in vitro* results. An understanding of protein-nanoparticle interactions resulting in PC formation would not only benefit toxicity assessment by *in vitro* approaches, but also their usefulness in human health applications. Recently we demonstrated through label-free quantitative mass spectrometry that carbon nanotubes with different surface functionalization acquire different PCs following incubation in typical cell culture media containing fetal bovine serum proteins [27]. These unique PCs likely influence the cellular effects and bioactivity of these nanotubes and are important factors to consider when comparing across studies.

In the current study, through the use of label-free quantitative mass spectrometry, we have characterized the formation of the PC on AgNPs of different sizes, 20 or 110 nm, and with different coatings, PVP or citrate. We utilized a comprehensive proteomics approach to determine the identities and abundance of proteins forming the PC on these AgNPs following incubation in commonly used cell culture media (DMEM) with 10% fetal bovine serum. Through the use of particles that share the same chemical composition, but vary based upon size and surface coating, insight can be gained regarding the effects of these characteristics on PC formation and, ultimately, its effects on AgNP-induced biological responses. Specifically, we examined differences in constituent or overall proteins found to associate with AgNPs (Section 2.2), and unique proteins which were found to associate only with a particular AgNP (Section 2.3).

## Results and Discussion

### 1. AgNP Characteristics

**Figure 1:**
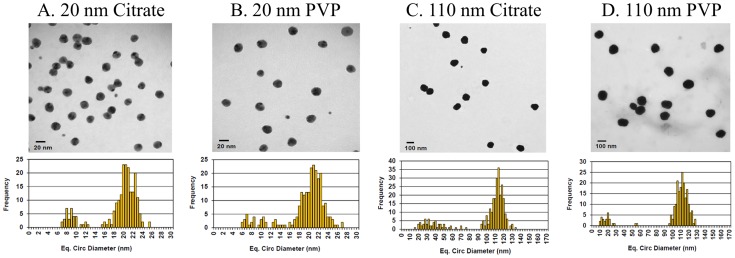
Scanning electron microscopy images and size distribution of A) 20 nm citrate-stabilized AgNP, B) 20 nm PVP-stabilized AgNP, C) 110 nm citrate-stabilized AgNP, and D) 110 nm PVP-stabilized AgNP samples confirming the diameters of all AgNPs used in this study.

Citrate- and PVP-stabilized AgNPs were characterized for hydrodynamic size and zeta potential in suspensions of water or DMEM cell culture media without serum (Table 1). Diameters of the AgNPs were confirmed via size distribution analysis by scanning electron microscopy (Figure 1). Citrate-stabilized AgNPs suspended in water were found to have more negative zeta potentials, compared to PVP-stabilized of similar diameter (Table 1). Furthermore, PVP-stabilized AgNPs were found to have a larger hydrodynamic size compared to citrate-stabilized particles of the same diameter. Characteristics of the AgNPs were found to change slightly when suspended in DMEM without serum (Table 1). 20 nm AgNPs in cell culture media were found to have a decreased hydrodynamic size compared to those suspended in water, whereas the hydrodynamic size of the 110 nm AgNPs were found to increase in cell culture media (Table 1). Citrate-stabilized AgNPs were also found to have lower zeta potentials compared to PVP-stabilized (Table 1). Overall, suspension of the AgNPs in different media exhibited minimal impact on hydrodynamic size, but did appear to influence the zeta potential which likely influences the association of proteins with the nanoparticle surface through electrostatic interactions.

**Table 1:** AgNP suspension characterization in water or serum-free cell culture media.

	Original Suspension (H_2_O)		Cell Culture Media (Serum-free)	
Nanomaterial	Hydrodynamic Size (nm)	Zeta Potential (mV)	Hydrodynamic Size (nm)	Zeta Potential (mV)
20 nm, AgNP-Citrate	24.4±0.4	−46.6±2.6	23.4±0.5	−25.3±1.4
20 nm, AgNP-PVP	26.6±0.6	−35.9±1.5	22.9±0.8	−27.9±0.8
110 nm, AgNP-Citrate	104.5±0.4	−43.6±1.4	120.9±12.9	−25.1±3.2
110 nm, AgNP-PVP	113.2±1.0	−25.5±1.1	121.5±1.2	−30.0±1.7

### 2. Proteomic Results Comparing Constituent Proteins of the PC

Nanoparticle bioactivity and toxicity is influenced through the addition of the PC in biological fluids by modifying the surface characteristics and thus the interface by which the cell interacts. The PC is a complex and dynamic entity which consists of two layers in general. The first layer is a hard corona which forms rapidly by the association of strongly bound proteins, which do not readily disassociate [28], [29]. The second layer is a soft corona, which is more variable, with proteins associating and dissociating almost constantly [28], [29]. In our current study to specifically examine the hard corona, which influences the activity and formation of the soft corona, particles underwent a series of washes to remove the soft corona. To comprehend the dynamic nature of the soft corona we must first understand the composition and interactions of the hard corona.

**Figure 2:**
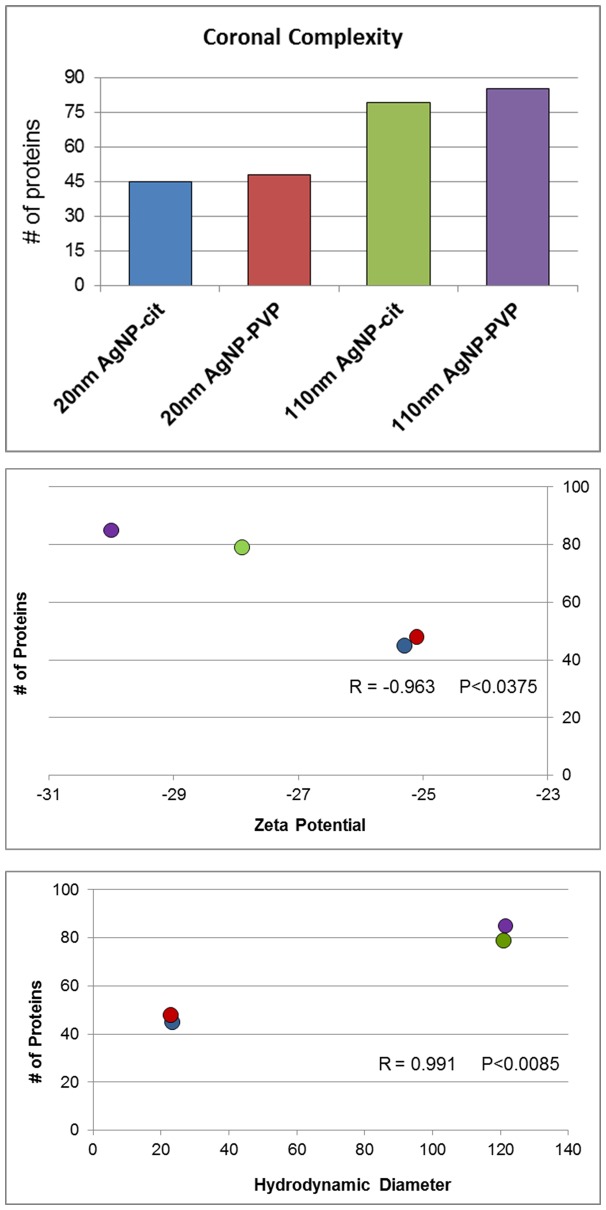
Total number of constituent and unique proteins found to associate with AgNPs after incubation in DMEM cell culture media containing 10% fetal bovine serum (2A). Samples were analyzed via HPLC-MS and proteins and peptides were identified using the UniProtKB Bos Taurus (Bovine) database and validated by PeptideProphet. Only proteins with a probability ≥0.9, or peptides with a probability ≥0.8, and a peptide weight ≥0.5 were used in the quantitation algorithm. Correlation of total number of constituent proteins found to associate with each AgNP and zeta potential (2B) or hydrodynamic diameter (2C).

Proteomic analysis by label-free quantitative mass spectroscopy identified and quantified 133 different protein components of the various AgNP-PCs. A complete list of these proteins, along with mass spectral and quantitation data, can be found in Tables S1 and S2, respectively. The number of constituent proteins detected in each AgNP corona is presented graphically in Figure 2A. AgNPs with a diameter of 110 nm were found to associate the largest number of constituent proteins compared to 20 nm AgNPs (Figure 2A). Such an observation may be explained in terms of the net reduction in Gibbs free energy (ΔG = ΔH-TΔS; ΔG, ΔΗ, and, ΔS are changes in Gibbs free energy, enthalpy, and entropy). It is well known that proteins adsorb on to nanoparticles by increasing their ΔS (or reducing ΔG) via perturbations in the protein’s secondary structure. Most importantly, the gain in ΔS is higher for larger particles since they provide larger areas for protein-surface interactions [30]. Consistently, the number of constituent proteins found to form each AgNP’s PC was also determined to be related to the hydrodynamic diameter. Specifically, the larger the hydrodynamic diameter of the AgNP the greater the number of individual constituent proteins found in the PC (Figure 2C). PVP-stabilized AgNPs were also found to associate a greater number of constituent proteins compared to citrate stabilized particles (Figure 2A). The number of total proteins found to bind each AgNP was determined to be related to the zeta potential, with the more negative the AgNP’s zeta potential the greater the number of amphiphilic proteins which were found to associate (Figure 2B), indicating the major role of electrostatic interactions in PC formation. By suspending AgNPs in media and altering the particle’s zeta potential, the diversity of the proteins forming the PC may be affected, likely through alterations in nonspecific electrostatic interactions with the proteins. Furthermore, a size dependent effect on the diversity of the PC may occur through differences in surface curvature which allows for dissimilar protein accumulation and selection.

Recently we identified 2,507 individual proteins, polypeptides, or protein fragments/isoforms in the 10% FBS-DMEM used in this study and determined individual peptide sequences and abundance data [27]. To evaluate characteristics of the constituent proteins which influence the formation of the PC, we assessed the hydropathicity (GRAVY), aliphatic index, and isoelectric point of all proteins and peptides detected for each AgNP-PC (Figure S1A in File S1). It was determined that the constituent proteins found to associate with 20 nm AgNPs were significantly more hydrophobic compared to the constituent proteins which associated with the 110 nm AgNPs. These findings suggest that hydrophobic interactions between the 20 nm AgNPs and the proteins may account for some of the differences in the identities of the proteins which bound to the different sized particles. No significant differences were determined for the other constituent protein/peptide parameters (e.g. isoelectric point, aliphatic index and cysteine content) assessed in this study. The aliphatic index computed for each individual protein is based on all of the charges of the amino acids present within a given protein but does not account for the conformation of the protein and the amino acids that are available for interaction with the nanoparticles. This means that our dataset may underestimate the importance of nonspecific electrostatic interactions and neglect the role of protein conformational changes by diluting differences in aliphatic indices. This led us to examine cysteine content of the constituent proteins since cysteine-nanoparticle interactions have been hypothesized to facilitate the association of proteins with nanomaterials and the conjunction of metals with the thiol group of cysteine is well-known [31]. No significant differences were determined between the PC that formed on AgNPs with respect to cysteine (Figure S1C in File S1), implying the absence of silver ion release in the hard corona obtained with thorough washing. Overall, the role of individual amino acids in the formation of the PC may best be assessed through individual protein-nanoparticle studies due to availability of the amino acids and the high number of peptide fragments found in typical fetal bovine serum [32], [33].

**Figure 3:**
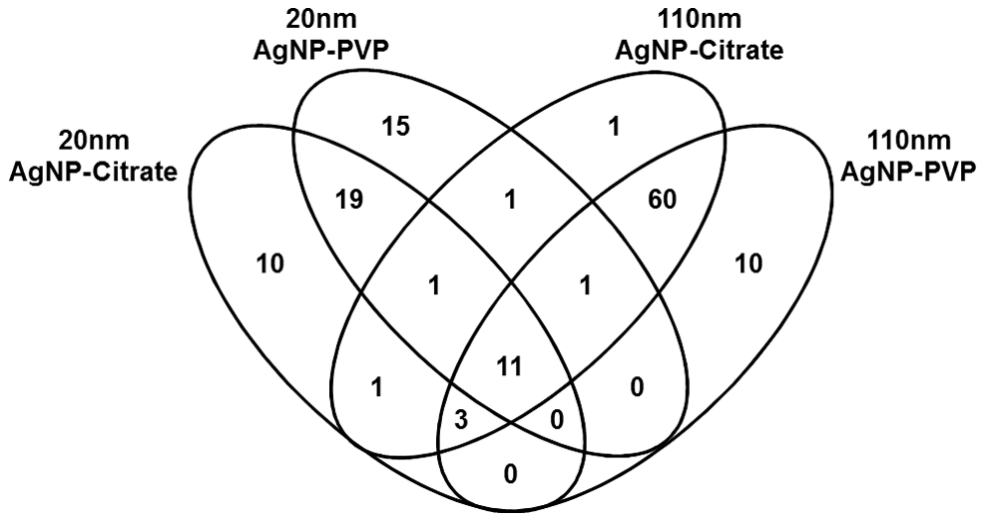
Venn diagram representing the distribution of proteins found to associate with AgNPs following incubation in DMEM cell culture media containing 10% fetal bovine serum. Eleven proteins were found to associate with all AgNPs.

**Figure 4:**
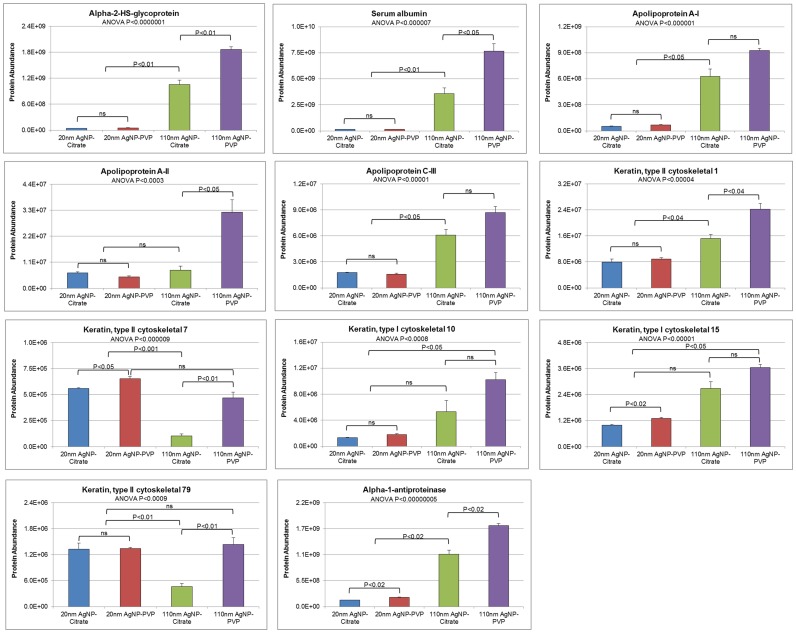
The individual abundance of each of the 11 common proteins found to associate with all AgNPs.

Many of the 133 proteins found to associate with AgNPs were shared; however, all AgNP-PC were found to share 11 common proteins (Figure 3). These proteins, which were common to all PCs included: alpha-1-antiproteinase, alpha-2-HS-glycoprotein, apolipoprotein A-I, apolipoprotein A-II, apolipoprotein C-III, keratin, type II cytoskeletal 1, keratin type II cytoskeletal 7, keratin type I cytoskeletal 10, keratin type I cytoskeletal 15, keratin type II cytoskeletal 79, and serum albumin. To determine differences in PC formation based on these commonly associated proteins, we assessed their individual abundance in each AgNP corona (Figure 4). In general, 8 of the 11 common proteins were determined to be more abundant on 110 nm AgNPs (PVP-stabilized>citrate-stabilized) compared to 20 nm AgNPs (Figure 4). Interestingly, apolipoprotein A-II although common to all PCs was significantly more abundant on 110 nm PVP-stabilized AgNPs compared to all other AgNPs (Figure 4), possibly due to its tubular structure, which requires less bending and structural coordination and ample hydrogen bonds along the protein peripherals to initiate contact with the PVP coating of the AgNPs. Furthermore, although common to all PCs, the keratin type II cytoskeletal proteins 7 and 79 were found to be significantly less abundant on 110 nm citrate-stabilized AgNPs, likely due to their neutral charge that favored the less polar backbone of the PVP. The suspension of nanomaterials is an important parameter regarding both their usefulness and the assessment of toxicity. To produce consistent suspensions, stabilizers are utilized to increase their solubility, e.g. citrate and PVP. Specifically, PVP-stabilized AgNPs have been shown to be more stable in water compared to citrate, likely due to steric repulsions arising from the large, non-charged PVP groups [34]. Our study demonstrates differences in the PC formation resulting from the use of these different stabilizers which may affect the functionality and/or toxicity of the nanomaterial. Since both PVP-and citrate-stabilized AgNPs were found to have similar hydrodynamic sizes it is unlikely that these differences in protein abundance are due to variations in aggregation state but due to interactions facilitated by the stabilization material. Also it is likely that citrate, based upon its smaller size, more thoroughly coats AgNPs compared to PVP, thus allowing less surface area for proteins to associate, and decreasing their abundance within the PC.

A comparison of the 20 most abundant proteins found to associate with each AgNP reveals that many are similar (Table 2 in bold). Interestingly, 4 common proteins (serum albumin, alpha-1antiproteinase, alpha-2-HS-glycoprotein, and apolipoprotein A-I) are in the top 6 most abundant for each AgNP. The most abundant protein found to associate with both 110 nm AgNPs and the 2^nd^ most for 20 nm AgNPs was serum albumin (Table 2 and Table S2). This is to be expected due to the high amount of serum albumin present in the media [27]. It is likely that many of the peptide fragments that were discovered to associate with the AgNPs were fragments associated with serum albumin itself and were not directly associated with the particles. Previously, we have characterized the FBS-DMEM media used in this study and demonstrated that there are 2,507 identifiable proteins/fragments, of which only 10 individual proteins account for more than 1% of total protein [27]. These ten most abundant include serum albumin (34.3%), alpha-2-HS-glycoprotein (6.6%), alpha-1-antiproteinase (3.3%), adenosine 3-phospho 5-phosphosulfate transporter 2 (2.5%), heat shock protein HSP 90 alpha (1.9%), nasal embryonic luteinizing hormone-releasing factor (1.7%), dynein (1.7%), DNA topoisomerase 2-binding protein 1 (1.2%), alpha-fetoprotein (1.1%), and WD repeat-containing protein 96 (1.0%). All other identifiable proteins/peptides represented less than 1% of the proteins present and the majority of total components (70%) within the FBS-DMEM were intracellular proteins. Many of the most abundant proteins/peptides associated with the AgNPs were found to be selectively enriched within the PC, meaning they represented less than 1% of the protein found within the FBS-DMEM (Table 2). This selective enrichment demonstrates the exclusivity by which proteins/peptides within typical cell culture media associate with particles. To understand if the individual characteristics of the proteins found to associate with the AgNPs were related to their abundance in the PC, we correlated the individual abundances of all constituent proteins within each PC and compared them to their hydropathicity, isoelectric point, and aliphatic index (Figures S2 and S3 in File S1). The protein characteristics assessed were found to be unrelated to their abundance within each AgNP’s PC. These findings further suggest that factors other than hydrophobicity may be responsible for PC formation such as nonspecific electrostatic interactions or hydrogen binding.

**Table 2:** 20 most abundant coronal proteins associated with each AgNP.

20 nm AgNP-citrate	110 nm AgNP-citrate	20 nm AgNP-PVP	110 nm AgNP-PVP
*Histone-lysine N-methyltransferase*	**Serum albumin**	**Alpha-1-antiproteinase**	**Serum albumin**
**Serum albumin**	**Alpha-1-antiproteinase**	**Serum albumin**	**Alpha-2-HS-glycoprotein**
**Alpha-1-antiproteinase**	**Alpha-2-HS-glycoprotein**	Apolipoprotein B-100	**Alpha-1-antiproteinase**
*PDZ domain-containing protein 2*	**Apolipoprotein A-I**	**Apolipoprotein A-I**	**Apolipoprotein A-I**
**Apolipoprotein A-I**	Serotransferrin	**Alpha-2-HS-glycoprotein**	Serotransferrin
**Alpha-2-HS-glycoprotein**	Alpha-2-macroglobulin	*Peptidylprolyl isomerase domain* *and WD repeat-containing protein 1*	Alpha-2-macroglobulin
Complement C3	Alpha-fetoprotein	40S ribosomal protein S12	Alpha-fetoprotein
Thrombospondin-1	Apolipoprotein B-100	*Kalirin*	Apolipoprotein B-100
Titin	Alpha-2-antiplasmin	**Keratin, type II cytoskeletal 1**	Complement C3
*Tight junction protein ZO-1*	Complement C3	Titin	Alpha-2-antiplasmin
40S ribosomal protein S12	Beta-2-glycoprotein 1	Spectrin beta chain	Inter-alpha-trypsin inhibitor heavy chain H1
*Zinc finger protein 469*	Fetuin-B	Low-density lipoproteinreceptor-related protein 1 intracellular domain	Fetuin-B
*Pericentrin*	Inter-alpha-trypsin inhibitor heavy chain H1	*Structural maintenance of* *chromosomes flexible hinge domain-containing protein 1*	Beta-2-glycoprotein 1
Spectrin beta chain	Hemoglobin fetal subunit beta	*Solute carrier family 25, member 44*	Hemoglobin fetal subunit beta
**Keratin, type II cytoskeletal 1**	Inter-alpha-trypsin inhibitor heavy chain H3	*Protein SOGA1*	Inter-alpha-trypsin inhibitor heavy chain H3
Vitronectin	Inter-alpha-trypsin inhibitor heavy chain H2	Poly [ADP-ribose] polymerase 1	Inter-alpha-trypsin inhibitor heavy chain H2
LDL receptor-related protein 1	Hemoglobin subunit alpha	**Apolipoprotein A-I**I	Vitamin D-binding protein
**Apolipoprotein A-I**I	Complement factor B	Transcription elongation factor SPT6	Transthyretin
*LDL receptor-related protein 12*	Hemopexin	*Coiled-coil domain containing 82*	Hemoglobin subunit alpha
BOD1L protein	Serpin A3–6	Dystonin	Complement factor B

Italized proteins are unique to that AgNP corona; **Bold** proteins are common to all AgNP coronas (from Figure 3).

### 3. Proteomic Results Comparing Unique Proteins of the PC

**Figure 5:**
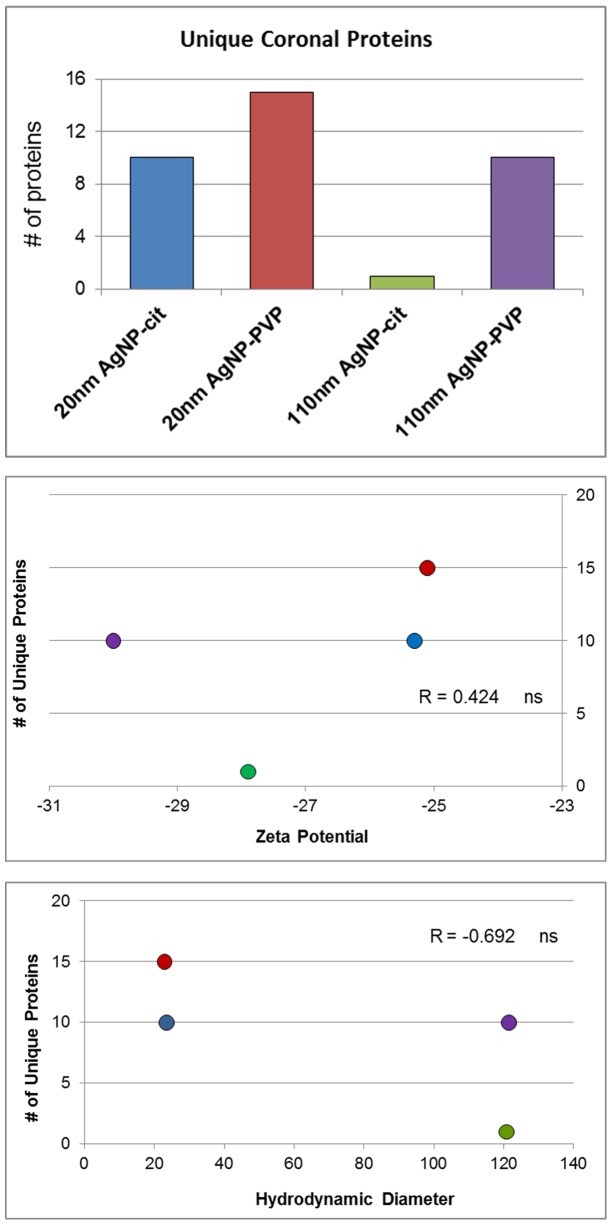
The number of unique proteins detected in each AgNP protein corona after incubation in DMEM cell culture media containing 10% fetal bovine serum (3A). Samples were analyzed via HPLC-MS and proteins and peptides were identified using the UniProtKB Bos Taurus (Bovine) database and validated by PeptideProphet. Only proteins with a probability ≥0.9, or peptides with a probability ≥0.8, and a peptide weight ≥0.5 were used in the quantitation algorithm. Correlation of the number of unique proteins found to associate with each AgNP and zeta potential (3B) or hydrodynamic diameter (3C).

Unique proteins which associated with the PC for each AgNP were assessed to determine distinctive characteristics of each individual PC. Even though 20 nm AgNPs were found to associate the fewest constituent proteins, compared to 110 nm AgNPs (Figure 2A), they were found to bind the greatest number of unique proteins (Figure 5A). PVP-stabilized AgNPs were also found to bind a higher number of unique proteins compared to citrate-stabilized AgNPs (Figure 5A). Table 3 provides a detailed list of the identities and the abundance of these unique proteins in the PC of each AgNP. Regarding abundance, many of the unique proteins, which only bound to a specific AgNP, were found to be highly abundant on 20 nm AgNPs (Table 2, and Table S2). The selective enrichment of these highly abundant unique proteins, which were found to only bind to 20 nm sized citrate- and PVP-suspended AgNPs and not 110 nm AgNPs (Table 2 italized) represent a list of previously unidentified proteins found to associate with AgNPs. This distinctive profile may influence the bioavailability and biological effects of these 20 nm AgNPs. Due to their high abundance and uniqueness histone-lysine N-methyltransferase, PDZ domain containing protein 2, and tight junction protein ZO-1 may be candidates as signature 20 nm citrate-suspended AgNP PC proteins whereas peptidylprolyl isomerase domain and WD repeat-contain protein 1 and kalririn may be signature 20 nm PVP-suspended AgNP PC proteins. The selective enrichment of these unique proteins on the 20 nm AgNPs demonstrates how the formation of the PC can be influenced by not just the particles chemical composition but also its size.

**Table 3:** Proteins Unique to Silver Nanoparticle Coronas.

	Protein ID	Gene Name	Protein Name	Quantity
**Reference Proteins** *	P34955	SERPINA1	Alpha-1-antiproteinase	789,162,341
	P15497	APOA1	Apolipoprotein A-I	419,360,364
	P02769	ALB	Serum albumin	2,884,521,505
**20 nm, citrate** **stabilized silver**	F1MLZ9	GRIN2A	Glutamate [NMDA] receptor subunit epsilon-1	2,839,052
	E1BN03	LRP12	Low-density lipoprotein receptor-related protein 12	5,816,234
	E1BC24	MDN1	Midasin	4,876,754
	F1MYZ3	MLL3	Histone-lysine N-methyltransferase	242,545,733
	E1BKZ0	PCNT	Pericentrin	13,363,362
	F1MT13	PDZD2	PDZ domain-containing protein 2	127,462,797
	G3N022	SPEG	Striated muscle preferentially-expressed protein kinase	4,513,921
	E1B9V7	WDR37	WD repeat-containing protein 37	4,115,664
	G3N2D0	ZNF469	Zinc finger protein 469	13,579,517
	F1N2D3	ZO1	Tight junction protein (Zona occludens 1) ZO-1	14,518,250
**20 nm, PVP** **stabilized silver**	E1BK41	ADAMTSL1	ADAMTS-like 1 (Punctin-1)	557,764
	E1BMW2	AP2A1	AP-2 complex subunit alpha-1	1,701,020
	F1MEZ4	CCDC82	Coiled-coil domain containing 82	4,096,417
	E1BK20	GCKR	Glucokinase regulatory protein	2,656,407
	F1MXJ6	KALRN	Kalirin	10,814,400
	A1L595	KRT17	Keratin, type I cytoskeletal 17	2,022,711
	A1A4K4	LLGL1	Lethal giant larvae homolog 1 (Drosophila)	945,511
	G5E5D5	PAXIP1	PAX-interacting protein 1	2,243,775
	Q29RZ2	PPWD1	Peptidylprolyl isomerase domain & WD repeat-containing protein 1	14,653,683
	F1MMG6	SCARA3	Scavenger receptor class A member 3	3,066,624
	A0JN83	SLC25A44	Solute carrier family 25, member 44	6,780,850
	F6RF21	SMCHD1	Structural maintenance of chromosomes flexible hinge domain-containing protein 1	7,334,983
	E1BLI8	SOGA1	Protein SOGA1	6,416,587
	G3MWW2	TET1	Methylcytosine dioxygenase	3,121,311
	G3N1S7	WDR52	WD repeat-containing protein 52	2,758,260
**110 nm, citrate stabilized silver**	F1MUY2	KRT6C	Keratin, type II cytoskeletal 6C	1,889,425
**110 nm, PVP** **stabilized silver**	Q3Y5Z3	ADIPOQ	Adiponectin	5,162,717
	F1MY85	C5	Complement C5	329,060
	F1MH27	CHIA	Chitinase, acidic mammalian	4,515,283
	P01035	CST3	Cystatin-C	1,030,016
	E1BCW0	HGFAC	Hepatocyte growth factor activator	5,656,261
	Q2KIU3	HP-25	Protein HP-25 homolog 2	7,221,263
	G5E5T5	IGHM	Ig mu chain C	209,449
	P07224	PROS1	Vitamin K-dependent protein S	5,426,924
	F1MPT4	SDK2	Protein sidekick-2	13,362,160
	A5PJ69	SERPINA10	Protein Z-dependent protease inhibitor	474,504

* found in all AgNP coronas, shown to emphasize quantitative differences between these and low-abundance AgNP-specific proteins; mean quantity shown.

In total, 19 proteins were found to uniquely bind to only 20 nm AgNPs (PVP and citrate) whereas 60 were found to only bind to 110 nm AgNPs (PVP and citrate) (Figure 3). These highly differential protein components may also provide a PC signature, which is based upon size and/or surface curvature of the AgNP. An examination of unique proteins based on the presence of PVP or citrate shows that only one unique protein, cumulus cell-specific fibronectin 1, bound to only citrate-stabilized AgNPs (20 and 110 nm), while no unique proteins were found bind to PVP-stabilized (Figure 3). These limited unique proteins identified between PVP- and citrate-stabilized AgNPs seems to suggest that although coatings may influence the abundance of proteins (Tables 2 and 3) they may not have much effect on the diversity of proteins forming the PC compared to size (Figures 2A and 3).

To assess how characteristics of the nanomaterial govern the formation of the PC, we analyzed the correlation between the number of unique proteins found to bind with the hydrodynamic sizes and zeta potentials of each AgNP (Figure 5B and 5C). We determined that the number of unique coronal proteins correlated best with hydrodynamic size, with the larger particles binding fewer unique proteins. This may be due to crowding out of other proteins because of the high abundance of large proteins, such as serum albumin (Table S2). Again, we assessed protein characteristics such as hydrophobicity, aliphatic index, and isoelectric point, which may influence the association of these unique proteins or peptide components with the AgNPs (Figure S1B in File S1). No significant differences in these evaluated protein characteristics were determined for these unique proteins in regard to their association with different AgNPs.

Recently, we examined the physiochemical interactions of a simplified PC by examining the association of an individual protein, bovine serum albumin, with 110 nm PVP- and citrate-stabilized AgNPs [33]. Similar to our present study, we found that serum albumin readily coated the particles and that PVP-stabilized AgNPs associated significantly more serum albumin compared to citrate-stabilized AgNPs. The association of serum albumin was also found to be governed by increased changes in protein conformation and to lesser extent hydrophobic interactions. This is similar to our current study which determined that 20 nm AgNPs bound more hydrophobic proteins compared to 110 nm AgNPs; however, associated significantly less serum albumin in terms of abundance. These findings suggest the importance of changes in protein conformation in the association of serum albumin and other proteins compared to hydrophobic interactions. In other words, the differences in complexity and abundance of the PC constituents may be also related to the free energy in protein folding and unfolding induced by the different surface groups for AgNPs of the same size, whereas the differences in PC formation for 20 and 110 nm AgNPs may be related to the surface curvature of the nanoparticles and the consequent energetics involved in protein adsorption and crowding.

In a recent study, we described the PC of a variety of carbon nanotubes utilizing the same label-free quantitative mass spectroscopy approach used in our current study [27]. Notably, there are differences between the constituents of PCs formed on the carbon nanotubes and AgNPs. Even though many proteins were shared, as expected due to high concentrations in FBS, (serum albumin, alpha-2-HS-glycoprotein, alpha-1-antiproteinase), a few were selectively enriched similarly (apolipoprotein AI, apolipoprotein AII, Keratin type 1 cytoskeletal protein 10, keratin type 1 cytoskeletal protein 15, and keratin type 2 cytoskelatal protein 1). Interestingly however, carbon nanotubes were found in general to selectively enrich different proteins (titin, alpha-S1-casein, keratin type 1 cytoskeletal protein 15 and keratin type 2 cytoskeletal proteins 5, 6A, 6C, and 75) whereas AgNPs uniquely associated apolipoprotein CIII, and keratin cytoskeletal proteins 7 and 79. Ultimately, these differences in protein composition may provide unique PC signatures between AgNPs and carbon-based nanomaterials which differ in curvature, chemical composition, hydrophobicity, surface coating and, notably, their stark contrast in initiating pi-stacking with the aromatic protein moieties.

It is likely that the PC on AgNPs and other nanomaterials may be significantly different based on the media they are suspended in. In the current study we utilized an extremely common cell culture media (DMEM) with 10% FBS. Other medias with different pH, salt concentrations, protein components, FBS concentrations, and other various characteristics may modify the individual constituents of the PC and their abundance within the PC. These alterations in the PC have been shown to alter the cellular uptake and activity of the nanomaterials [35], [36], [37]. Specifically, Lesniak et al. demonstrated differences in uptake when cells were exposed to polystyrene nanoparticles, which were incubated with serum with or without heat inactivation [35]. This study determined that cells more readily internalized polystyrene nanoparticles after incubation with serum that had not undergone heat inactivation compared to heat inactivated serum. Removal of complement from serum by heat inactivation reduced cellular uptake demonstrating that available proteins, which associate with the PC, can affect the fate of the nanoparticle [35]. Furthermore, research has demonstrated differences in the formation of a PC as related to the protein concentrations present [29]. These findings suggest that there are significant differences between the formation of the PC on a particle at protein concentrations seen under *in vitro* conditions and *in vivo*. These variations in conditions resulting in differential PC formation are likely to cause variability in the formation of the PC in individuals suffering from diseases treated with nanomedicines. Specifically these disease states may modify both serum chemistry and concentrations of individual proteins thus altering the PC and the therapeutic applications of the nanomedicine.

## Conclusions

The implications of the PC on the eventual activity and fate of intravenously injected nanomaterials and the assessment of nanomaterials via *in vitro* toxicity testing are immense. In our current study, we have described the differences in the diverse protein composition between particles of identical chemical composition that differ based on size (20 and 110 nm) and coating (PVP and citrate). The interactions and association of proteins with the surface of the nanomaterials may also induce conformational changes to the proteins resulting in toxicity through modified immune responses. It is likely that these interactions with protein may also modify AgNP dissolution and thereby affect toxicity [38]. Furthermore, it is likely that the PC may protect cells from the nanomaterial until the PC is degraded in the lysosomes resulting in lysosomal damage and ultimately apoptosis [39]. It is likely in the case of AgNPs that the removal of the PC in phagocytic cells may result in the release of toxic Ag^+^ leading to cell death. Ultimately, through a thorough understanding of the formation and composition of these PCs we may be able to mitigate toxicity and selectively utilize the PC to increase the therapeutic applications of nanomaterials.

## Methods

### Reagents and Materials

dl-Dithiothreitol (DTT), urea, triethylphosphine, iodoethanol, and ammonium bicarbonate were purchased from Sigma-Aldrich (St. Louis, MO, USA). LC-MS grade 0.1% formic acid in acetonitrile and 0.1% formic acid in water were purchased from Burdick & Jackson (Muskegon, MI, USA). Modified sequencing grade porcine trypsin was obtained from Princeton Separations (Freehold, NJ, USA). Dulbecco’s Modified Eagle’s Medium (DMEM) with glutamax and 10% heat inactivated fetal bovine serum were purchased from Invitrogen (Carlsbad CA). AgNPs were obtained from nanoComposix (San Diego, CA) by the NIH Nanocharacterization Laboratory.

### AgNP Characterization

The hydrodynamic size (Nanosizer S90, Malvern) and zeta potential (ZetaSizer Nano, Malvern) of all AgNPs were characterized in DI water and DMEM culture media without fetal bovine serum (FBS). Scanning electron microscopy was utilized to verify the diameters of the AgNPs.

### Protein Corona Generation and Proteomic Characterization

Using a modification of Tenzer’s method, 1 mg of each AgNP type was suspended in 10 mL of DMEM culture media supplemented with 10% FBS, briefly sonicated in a bath sonicator, diluted 1∶10 in FBS/media, and incubated for 1 h at 37°C (to simulate *in vitro* exposure protocols) [40]. Stable PCs are at equilibrium within 5 min [41]. The samples were centrifuged (15 min at 3,000×g/22°C) and the pellets containing the AgNP-protein complexes were washed and pelleted three times with PBS. After the third and final wash, the supernatant was free of protein. PCs were solubilized *in situ* using a lysis buffer specific for label-free quantitative mass spectrometry (LFQMS) (8 M urea, 10 mM DTT freshly prepared). For comparative reference purposes, 100 µg of FBS supplemented culture media proteins were also solubilized for LC-MS/MS analysis. Briefly, protein samples were reduced and alkylated by triethylphosphine and iodoethanol and proteolyzed using porcine trypsin [42]. Exactly 20 µg of each tryptic digest sample was injected randomly as two technical replicates onto a C18 reversed phase column for a 3 h HPLC gradient separation, electrospray ionization, and analysis using an LTQ-PROTEOMEX ion trap mass spectrometer. A blank was injected between each sample to clean and balance the column, as well as, eliminate carryover. The acquired data were searched against the most up-to-date UniProtKB *Bos taurus* (Bovine) database using SEQUEST (v. 28 rev. 12) algorithms in Bioworks (v. 3.3). Peptide and protein identifications were validated by PeptideProphet and ProteinProphet in the Trans-Proteomic Pipeline (TPP, v. 3.3.0) [43], [44]. Only proteins and peptides with (a) protein probability ≥0.9, (b) peptide probability ≥0.8, and (c) peptide weight ≥0.5 were used in the quantitation algorithm. Identified bovine proteins whose names appeared as “uncharacterized” were annotated using homologous human proteins identified by UniProt Blast based on similarity in amino acid sequence.

Protein abundance was determined using IdentiQuantXL™ [45]. After chromatogram alignment and peptide retention time determination, a weighted mean m/z of each peptide is calculated and a tab delimited file was created to extract peptide intensity using MASIC [46]. Peptides were then filtered according to intensity CV across all samples and intensity correlation for those identifying a particular protein. Protein abundance (intensity) was calculated from all qualified peptides corresponding to a particular protein. Protein abundance/quantity calculated by this method are represented by unitless numerical values. Comparison of the mean abundance of individual protein in each PC, generated by LFQMS, was performed within the IdentiQuantXL™ platform using one-way ANOVA and Pairwise Multiple Comparisons (Holm-Sidak method). False discovery rate (FDR) was estimated using Q-value software (Storey et al. 2002).

### Protein Hydropathicity and Aliphatic Index Analysis

Grand average of hydropathicity (GRAVY) scores, aliphatic indices, and cysteine content for all identified proteins and peptides were calculated using the Protein Identification and Analysis Tools (ProtParam) on the ExPASy Server (http://web.expasy.org/protparam/) (Gasteiger et al. 2005). The GRAVY score for a peptide or protein is calculated as the sum of hydropathy values of all the amino acids, divided by the number of residues in the sequence. The aliphatic index of a protein is defined as the relative volume occupied by aliphatic side chains (alanine, valine, isoleucine, and leucine) and may be regarded as a positive factor for increased thermostability of globular proteins.

## Supporting Information

Table S1 **Identification of all proteins found to associate in the corona of each AgNP by label-free mass spectroscopy.**
(PDF)

Table S2 **Quantification of all proteins and peptides found to associate in the corona of each AgNP by label-free mass spectroscopy.**
(PDF)

File S1 This file contains Figures S1 through S3. Figure S1, Evaluation of characteristics of constituent protein components found to associate with each AgNP after incubation in DMEM containing 10% fetal bovine serum. Average GRAVY score, isoelectric point, and aliphatic index for constituent proteins and peptides (1A). Average GRAVY score, isoelectric point, and aliphatic index for unique proteins and peptides (1B). Evaluation of cysteine residues present in constituent and unique proteins and peptides (1C). Figure S2, Correlation of the characteristics of individual protein components to protein quantity in the protein corona formed on 20 nm citrate and PVP suspended AgNPs. Characteristics correlated include GRAVY score, isoelectric point, and aliphatic index. Figure S3, Correlation of the characteristics of individual protein components to protein quantity in the protein corona formed on 110 nm citrate and PVP suspended AgNPs. Characteristics correlated include GRAVY score, isoelectric point, and aliphatic index.
(PPTX)
