# Supplementary material for: Silver Nanoparticle Protein Corona Composition in Cell Culture Media
Source: PLoS One. 2013 Sep 9;8(9):e74001. doi: 10.1371/journal.pone.0074001 (PMC3767594; doi:10.1371/journal.pone.0074001)
Supplement: Table S1 — Identification of all proteins found to associate in the corona of each AgNP by label-free mass spectroscopy. (PDF) [file pone.0074001.s001.pdf]

Protein ID	Protein Entry	Gene Name	Protein Name	Protein Probability	Protein Coverage (%)	# of Unique Sequences	# of Identified Peptides
					Peptide Sequence	Peptide Charge	Peptide Probability
A0JN83	A0JN83_BOVIN	SLC25A44	Solute carrier family 25, member 44	99.9%	8.6	2	2
					GNPEGQGVVAFGQTK	3	99.9%
					VSVYPFTLIRTR	3	86.4%
A1A4K4	A1A4K4_BOVIN	LLGL1	Lethal giant larvae homolog 1 (Drosophila)	99.9%	3.67	2	2
Q8MKF0	L2GL1_BOVIN	LLGL1	Lethal(2) giant larvae protein homolog 1	99.9%	3.86	2	2
E1BMF9	E1BMF9_BOVIN	LLGL1	Uncharacterized protein	99.9%	3.77	2	2
					QELFAFHKTVEHGFPNQPSALAFDPELR	3	99.9%
					TVEHGFPNQPSALAFDPELRIMAIGTRSGAVK	3	99.9%
A1L595	K1C17_BOVIN	KRT17	Keratin, type I cytoskeletal 17	99.9%	6.12	2	2
					ADLEMQIENLKEELAYLR	3	99.9%
					VLDELTLAR	2	93.8%
A2I7M9	SPA32_BOVIN	SERPINA3-2	Serpin A3-2	100.0%	36.01	13	18
					DTQSIIFLGK	2	99.9%
					FIEDAQVLYSSEAFPTNFR	2	100.0%
					FIEDAQVLYSSEAFPTNFR	3	100.0%
					GSTLTEILEGLK	2	100.0%
					LLDKFIEDAQVLYSSEAFPTNFR	2	100.0%
					LLDKFIEDAQVLYSSEAFPTNFR	3	100.0%
					RIHELYLPK	2	99.8%
					RIHELYLPK	3	100.0%
					RVDGHTLASSNTDFAFSLYK	3	100.0%
					SLINDYVK	2	94.6%
					SNYELNDILSQLGIR	2	100.0%
					SNYELNDILSQLGIR	3	100.0%
					TELVLVNYIYFK	2	100.0%
					TQGKIEELFK	2	86.4%
					TVEVPMMTLDLETPYFR	2	100.0%
					VDGHTLASSNTDFAFSLYK	2	100.0%
					VDGHTLASSNTDFAFSLYK	3	100.0%
					VNRPFLIAVVLK	3	100.0%
Q3ZEJ6	SPA33_BOVIN	SERPINA3-3	Serpin A3-3	100.0%	7.79	3	4
A2I7N0	SPA34_BOVIN	SERPINA3-4	Serpin A3-4	100.0%	7.79	3	4
					GPTLTEILEGLK	2	99.8%
					RVDGHTLASSNTDFAFSLYK	3	100.0%
					VDGHTLASSNTDFAFSLYK	2	100.0%
					VDGHTLASSNTDFAFSLYK	3	100.0%
A2I7N3	SPA37_BOVIN	SERPINA3-7	Serpin A3-7	100.0%	8.39	3	3
					DAEAFYASEVLSTNFK	2	100.0%
					DILSQLGIK	2	99.7%
					FSISSHYQLK	2	96.4%
					KNGATPFIIAGIVGNVKLLK	3	99.9%
A5PJ69	A5PJ69_BOVIN	SERPINA10	SERPINA10 protein	100.0%	8.19	2	2
					IFLPWADLSDLMVTER	2	100.0%
					IGDHLTLEDYLTTDLVDTWLR	3	99.9%
A5PJE3	A5PJE3_BOVIN	FGA	Fibrinogen alpha chain	100.0%	6.67	3	3
P02672	FIBA_BOVIN	FGA	Fibrinogen alpha chain	100.0%	6.67	3	3
					GLIDEVDQDFTSR	2	89.0%
					GNLDDFFHR	2	99.8%
					TGLAPEFAALGESGSSSSK	2	100.0%
A6QNZ7	A6QNZ7_BOVIN	KRT10	Keratin 10 (Epidermolytic hyperkeratosis; keratosis p	100.0%	7.6	4	4
P06394	K1C10_BOVIN	KRT10	Keratin, type I cytoskeletal 10	100.0%	7.6	4	4
					IRLENEIQTYR	2	94.0%
					LAADDFR	2	96.1%
					QSLEASLAETEGR	2	100.0%
					VLDELTLTK	2	99.8%
A6QPP2	A6QPP2_BOVIN	SERPIND1	SERPIND1 protein	100.0%	13.51	4	4
					AADFSDPAFLAHTNR	3	84.7%
					EYYFAEVR	2	92.9%
					HQGTITVNEEGTQAAAVTAVGFMPLSTQVR	3	99.9%
					LNLLNAQFAFDLYR	2	100.0%
A7E350	A7E350_BOVIN	PLG	PLG protein	100.0%	4.56	2	2
					NYGGTVAVTESGHTCQR	3	100.0%
					TPENYPNAGLTMNYCR	2	100.0%
B8Y9T0	B8Y9T0_BOVIN	FN1	Cumulus cell-specific fibronectin 1 transcript variant	100.0%	2.25	3	3
B8Y9S9	B8Y9S9_BOVIN	FN1	Embryo-specific fibronectin 1 transcript variant	100.0%	2.14	3	3
P07589	FINC_BOVIN	FN1	Fibronectin	100.0%	2.06	3	3
G5E5A9	G5E5A9_BOVIN	FN1	Uncharacterized protein	100.0%	2.06	3	3
					SYTITGLQPGTDYK	2	89.6%
					TEIDKPSQMQVTDVQDNSISVR	3	99.9%
					VPGTSASATLTGLTR	2	98.0%
F1MJB7	F1MJB7_BOVIN	Bt.306	Uncharacterized protein	100.0%	14.44	9	9
P06868	PLMN_BOVIN	PLG	Plasminogen	100.0%	14.41	9	9
E1B726	E1B726_BOVIN	PLG	Uncharacterized protein	100.0%	14.41	9	9
					DCQAWDSQSPHAHGYIPSK	3	96.5%
					EAHLPVIENK	2	97.9%
					GTSSTTITGR	2	99.4%
					NYGGTVAVTESGHTCQR	3	100.0%
					TPENYPNAGLTMNYCR	2	100.0%
					VILGAHNEK	2	97.8%
					VREQSVQEIPVSR	2	96.7%
					VSPYVPWIEETMR	2	99.9%
					WSATSPHVPK	3	97.2%
E1B7E5	E1B7E5_BOVIN		40S ribosomal protein S12	99.9%	25.76	2	2
					PSKVVGCIYVVGK	3	99.9%
					TGGGLCAIHQINAIRADDNKK	3	99.9%
E1B8H1	E1B8H1_BOVIN	LOC784932	Uncharacterized protein	100.0%	19.9	9	9
					DAEAFYASEVLSINFK	2	95.6%
					DILSQLGIK	2	99.7%
					DILSQLGIKK	2	98.8%
					FSISSDYQLR	2	100.0%
					LAVSQVIHK	2	97.2%
					LINEYVK	2	96.2%
					VKVPMMTLR	3	98.3%
					VPMMTLR	2	94.6%

					VTSVDGHSLASSNTDFAFSLYK	3	100.0%
E1B990	E1B990_BOVIN	Bt.29236	Uncharacterized protein	100.0%	5.75	2	2
Q29S21	K2C7_BOVIN	KRT7	Keratin, type II cytoskeletal 7	100.0%	4.94	2	2
					LALDIEIATYR	2	99.9%
					SLDLDSIIAEVK	2	99.9%
E1B9V7	E1B9V7_BOVIN	WDR37	Uncharacterized protein	97.7%	6.45	2	2
					LPSSVRSTLLELFGQIER	3	85.1%
					STLLELFGQIEREFENLYIENLELRR	3	84.9%
E1BA03	E1BA03_BOVIN	PAK6	Uncharacterized protein	99.9%	6.31	2	2
					LLLDSYLKIGEGSTGIVCLAREK	3	99.9%
					RLFRSMFLSAPATAPPSSSK	3	99.9%
E1BC24	E1BC24_BOVIN	Bt.46644	Midasin	100.0%	0.55	2	2
					ELQYSTGHLVSADLSSR	3	81.8%
					REINKATDDFTTWK	3	99.9%
E1BCK9	E1BCK9_BOVIN	SNAPC4	Uncharacterized protein	99.9%	3.43	2	2
					EKALPEDPETCLQLNMVYQEVVREK	3	99.9%
					KAWPPALLQAGAGDPLPLQVRTER	3	99.9%
E1BCW0	E1BCW0_BOVIN	Bt.47625	Uncharacterized protein (Fragment)	97.6%	7.74	2	2
					TTDVTQTFGIEK	2	94.8%
					VANYVDWINDR	2	97.6%
E1BEB8	E1BEB8_BOVIN	SUPT6H	Uncharacterized protein	99.9%	2.26	2	2
					EYVEPELHINDLWRVWQWDEK	3	99.9%
					RDMYTICQSAGLDGLAKK	3	99.9%
E1BF81	E1BF81_BOVIN	Bt.17574	Uncharacterized protein	100.0%	7.43	2	2
					HYYELEALTTDFQDWAGASR	3	98.6%
					MDSVITALSR	2	98.1%
E1BFM5	E1BFM5_BOVIN		Uncharacterized protein	99.9%	1.8	2	2
F1MGK2	F1MGK2_BOVIN		Uncharacterized protein	99.9%	1.73	2	2
G3MWW2	G3MWW2_BOVIN		Uncharacterized protein	99.9%	1.77	2	2
					AALKVTSEGNPSIQLEDLGSR	3	99.9%
					LTVPVRSLLTRAGAAR	3	99.9%
E1BGJ0	E1BGJ0_BOVIN	Bt.854	Uncharacterized protein	100.0%	0.97	2	2
					MLTPPLLLLLPLLSALVAAAVDVPKTCSPK	3	99.9%
					SLDPFKPFIIFSNR	3	99.9%
E1BGL8	E1BGL8_BOVIN	Bt.59431	Uncharacterized protein	99.9%	2.8	2	2
					LLISPAAEVAEGQVVTLSCR	3	99.9%
					WLQEGPEASLSFPVVTRAHAGAYTCQVR	3	99.9%
E1BH06	E1BH06_BOVIN	Bt.105154	Uncharacterized protein	100.0%	9.65	11	11
					AAGLAFSDGDHR	2	99.8%
					AELADQAASWLTR	2	100.0%
					DSSTWLTAFVLK	2	99.9%
					FGLLGEDGEK	2	92.9%
					GSFDFPVGDAISK	2	99.9%
					ILSLAQDQVGGSAEK	2	99.6%
					LEEELQFSLGSK	2	99.9%
					LQDAPSGQVVR	2	99.9%
					LTSLSDRYVSHFETEGPHVLLYFDSVPTSR	3	87.3%
					QTDMQGVNLLFSSR	2	100.0%
					VTASDPLEALGSEGALSPGGLASLLR	2	87.9%
E1BI82	E1BI82_BOVIN	Bt.52938	Uncharacterized protein	99.8%	6.59	3	3
					GYYVVAVVK	2	92.7%
					HPTVLQNTDGKNPEAWAK	3	99.2%
					YLGPEYLQAIANVK	2	99.8%
E1BI98	E1BI98_BOVIN	COL6A1	Uncharacterized protein	99.9%	3.51	3	3
					AAEYDVVFGER	2	97.1%
					IALVITDGR	2	91.6%
					VAVVQYSGTGQQRPER	3	92.8%
E1BJV5	E1BJV5_BOVIN	ZNF638	Uncharacterized protein	100.0%	1.58	2	2
					ESGLLKPTSARSGVAENSSK	3	99.9%
					HKQNTEKFMAK	3	99.9%
E1BK20	E1BK20_BOVIN	GCKR	Uncharacterized protein	99.9%	6.34	2	2
					SRSTTNILLEALLPAAHKTVDHCR	3	99.9%
					TVDHCRWGIAASPR	3	99.9%
E1BK41	E1BK41_BOVIN	Bt.63004	Uncharacterized protein	99.9%	2.78	2	2
					CMYTPKMPTVQPCNIFDCPK	3	99.9%
					LQECNLDPCPASDGYKQIMPYDIYHPLPR	3	99.9%
					TGERSYECSECGKSFR	3	99.9%
E1BKZ0	E1BKZ0_BOVIN	PCNT	Uncharacterized protein	100.0%	1.14	2	2
					EVESLTQEQSEARRQADK	3	81.2%
					LRQLELGLTGDESLR	3	99.9%
E1BLI8	E1BLI8_BOVIN	Bt.60089	Uncharacterized protein	99.9%	3.15	2	2
					AEDNDSYASEIKELQLVLAEAHDSLR	3	99.9%
					AREAEALPGLREQAVLVSK	3	99.9%
E1BMJ0	E1BMJ0_BOVIN	SERPING1	Uncharacterized protein	100.0%	26.92	9	12
					AFMSEGFTSFSQIFHSSDLTIK	3	99.8%
					FHPTHLTMPR	3	99.9%
					FPVFMGR	2	91.4%
					HHLQDLEQALSTAVFK	2	98.9%
					HHLQDLEQALSTAVFK	3	97.3%
					HQATLELTESGVEATAASVVSVAR	2	99.8%
					HQATLELTESGVEATAASVVSVAR	3	97.8%
					LILLNAVALSAK	2	100.0%
					LLDSLPEDTR	2	99.8%
					LYQDFSVLK	2	98.6%
					SAEAVLGEALTDFSLR	2	100.0%
					SAEAVLGEALTDFSLR	3	100.0%
E1BMW2	E1BMW2_BOVIN	AP2A1	Uncharacterized protein (Fragment)	99.9%	3.93	2	2
					GPGAGSALDDSR	3	99.9%
					QIGYLFISVLVNSNSELIRLINNAIK	3	99.9%
E1BN03	E1BN03_BOVIN	LRP12	Uncharacterized protein	99.9%	5.01	2	2
					CDGYWHCPNGRDEINCTVCQK	3	99.9%
					GGHTETGRDVTSVEPPSVSPAR	3	99.9%
E1BNR0	E1BNR0_BOVIN	Bt.23652	Uncharacterized protein	100.0%	15.04	50	51
					ALQEATYQTPDFIVPLTDLR	2	100.0%
					AQLSDPTLNPLVLK	2	100.0%
					AQSVYQELLAQEDR	2	100.0%
					ATGALYDYVNK	2	99.9%
					ATLRIARDGMSTSATTSLK	3	100.0%
					ATSNFPVDLSAFPK	2	90.1%
					DEVLENVSPSCPK	2	100.0%

					DSVGQPQEFTLVASVK	2	92.0%	
					ELSELSLPEFK	2	98.3%	
					EVLLQTFLDDTSPGDKR	2	95.2%	
					FELELKPTGEVEQYSAK	3	100.0%	
					FFEEGTEAVGLAFESTR	2	100.0%	
					FSALDMTNNGK	2	98.4%	
					FSVPAGILVPSFGTLTAR	2	99.8%	
					GFEPTLEALFGK	2	99.9%	
					GLQDSADQVYQGAMR	2	100.0%	
					GNVATEISIER	2	99.3%	
					GTTETYQQWK	2	99.0%	
					HVSEAICNEQHLFLPFSYK	3	92.6%	
					ILGEELGFVK	2	99.8%	
					INDEIQALEFPQK	2	99.4%	
					IPGYTIPVANIEVSPFTVK	2	100.0%	
					IPSFQINFK	2	99.3%	
					ITELSTSAQEVIK	2	97.1%	
					ITQLLSGEQNEQVK	2	95.5%	
					KITEVTLTGHISYDR	3	83.0%	
					KMELSDEVR	2	97.4%	
					LAAYLMLMGSPSQSDISK	2	100.0%	
					LATALSLSNR	2	99.6%	
					LSQQVSDYLSTFNWER	2	100.0%	
					LSSVLQQVK	2	94.0%	
					LVAAASTWLQETSR	2	100.0%	
					MAEIVEK	2	97.3%	
					MELPNFHIPDNLFLK	3	92.0%	
					NFVASHIANILNSEDLYVQDLK	3	100.0%	
					NSMEIEIPLPFGGK	2	99.9%	
					NVDASVTTTAR	2	99.6%	
					QQVEAVDVR	2	99.6%	
					QVLLYPEKEEPK	2	96.9%	
					QVLLYPEKEEPK	3	91.9%	
					RVPQTDLTFR	2	98.2%	
					SLQDIFQEIESELKR	3	99.9%	
					SLQEYLSILANAEGR	2	97.8%	
					TMEQLTPK	2	85.0%	
					VAPGEFTITF	2	96.0%	
					VFGFASADLFEIGLEGK	2	100.0%	
					VLELDDEVQIK	2	99.8%	
					VSDSLIGVTQGYSVTVK	2	100.0%	
					VVVIGALETVQK	2	100.0%	
					YGMMAQVTQTLK	2	99.9%	
					YIVGATDFASQLSSQIEPLIQK	3	98.0%	
E1BNU3	E1BNU3_BOVIN	Bt.105935	Uncharacterized protein	99.9%	6.17	2	2	
					IDHQNRAGYSALMLAALTSVGREEDMAVVQR	3	99.9%	
					LELAQAQEGAPSPARAAPR	3	99.9%	
F1MC11	F1MC11_BOVIN	Bt.91072	Uncharacterized protein	100.0%	4.4	2	2	
					ALEEANADLEVK	2	99.9%	
					VLDELTLAR	2	93.8%	
F1MC45	F1MC45_BOVIN	Bt.68981	Uncharacterized protein (Fragment)	100.0%	3.06	2	2	
					EAFTMIGPR	2	98.6%	
					IENGFLSESTFTYPLNK	2	100.0%	
F1MCF8	F1MCF8_BOVIN	Bt.106009	Uncharacterized protein	100.0%	14.53	2	2	
					SPPSVTLFPPSTEELNGNK	2	99.3%	
					YAASSYLSLTSSDWK	2	100.0%	
F1MEZ4	F1MEZ4_BOVIN	Bt.57634	Uncharacterized protein	99.9%	7.09	2	2	
					ISSVMCDSDESDDSDILVR	3	99.9%	
					QKSYAQDMLTSLYYLDNR	3	99.9%	
F1MGQ6	F1MGQ6_BOVIN	KRT6A	Uncharacterized protein	100.0%	2.98	2	2	
					FASFIDK	2	82.0%	
					WTLLQEQGTK	2	98.7%	
F1MGW0	F1MGW0_BOVIN	Bt.23316	Uncharacterized protein (Fragment)	99.9%	2.35	2	2	
P02453	CO1A1_BOVIN	COL1A1	Collagen alpha-1(I) chain	99.9%	2.26	2	2	
					GNSGEPGAPGSK	3	99.9%	
					GPAGPQGPRGDKGETGEQGDR	3	99.9%	
F1MH27	F1MH27_BOVIN	CHIA	Uncharacterized protein	100.0%	6.57	2	2	
					ADGLYPVADNR	2	96.6%	
					FLHQYGFDGLDFDWEYPGSR	3	99.9%	
F1MI18	F1MI18_BOVIN	Bt.14949	Uncharacterized protein	100.0%	10.49	10	11	
F1MJK3	F1MJK3_BOVIN	Bt.14949	Uncharacterized protein	100.0%	10.47	10	11	
					AESLVFVQTDKPIYKPGQTVQFR	3	99.8%	
					AISYLISGYQR	2	98.9%	
					GLPFFGQVLLVNEK	2	99.0%	
					HSDGSYSTFGDR	2	97.9%	
					IEHSFEVDEYVLPK	2	99.4%	
					IEHSFEVDEYVLPK	3	100.0%	
					LLLQEVALPEIPGK	2	99.9%	
					LPSNVVEGSAR	2	98.8%	
					LQGGLSQLSFPLSVEPALGPYK	2	100.0%	
					VPHFPEIGQK	3	82.7%	
					VREEGTGLELTGHGSSEITNTLSK	3	100.0%	
Q3SX14	GELS_BOVIN	GSN	Gelsolin	100.0%	17.92	9	10	
F1MJH1	F1MJH1_BOVIN	GSN	Uncharacterized protein	100.0%	17.92	9	10	
					AGALNSNDAFVLK	2	99.9%	
					AQPVQVAEGSEPDSFWEALGGK	2	100.0%	
					HVVPNEVVVQR	2	99.9%	
					HVVPNEVVVQR	3	96.1%	
					NWRDPDQTDGPGLSYLSSHIANVER	3	99.9%	
					QTQVSVLPEGGETPLFK	2	98.4%	
					TASDFISK	2	92.3%	
					TGALELLR	2	98.4%	
					TPSAAYLWVGAGASEAEK	2	100.0%	
					YIETDPANR	2	99.4%	
F1MKE9	F1MKE9_BOVIN	Bt.27241	Uncharacterized protein (Fragment)	99.9%	1.5	2	2	
					KKHEAIETDTAAYEER	3	99.9%	
					QIETQEKPRDVSSVELLMK	3	86.5%	
F1MLW7	F1MLW7_BOVIN	Bt.106009	Uncharacterized protein	100.0%	20.94	3	3	
					SPPSVTLFPPSTEELNGNK	2	99.3%	
					VLTQPSSVSGSLGQR	2	100.0%	

					YAASSYLSLTSSDWK	2	100.0%
F1MLZ9	F1MLZ9_BOVIN	GRIN2A	Uncharacterized protein (Fragment)	99.9%	4.49	2	2
					APHGPSFTIGKAIWLLWGLVFNNSVPVQNPK	3	99.9%
					HSLPSQAANDGYLRSSLR	3	99.9%
F1MMD7	F1MMD7_BOVIN	Bt.5945	Uncharacterized protein	100.0%	30.46	21	25
Q3T052	ITIH4_BOVIN	ITIH4	Inter-alpha-trypsin inhibitor heavy chain H4	100.0%	30.46	21	25
					AAAQEQYSAAVAR	2	100.0%
					EATFQMELPK	2	98.8%
					ELKLDYQESPPGK	2	94.9%
					EQSPDVLLAQIR	2	99.8%
					ETLYSVMPGLK	2	99.5%
					FAHTVITSR	2	99.1%
					FSSHVSGTLGQFYQDVLWGPLDTADDSKR	3	100.0%
					GSELVVAGK	2	98.9%
					HLGAYELLLK	2	100.0%
					KTEQFQVSVSVAPAAK	2	100.0%
					KTEQFQVSVSVAPAAK	3	99.6%
					LLNGTPLFGPPGPPAAASPFHR	3	99.9%
					LREQSPDVLLAQIR	2	98.6%
					LREQSPDVLLAQIR	3	99.9%
					MALENGGLAR	2	99.6%
					NVIFVIDK	2	94.1%
					RPQELVNLLSDPDQGVEVTGHFETAK	3	100.0%
					SYATGIQAQGGTNINDAMLMAVQLLEK	2	100.0%
					SYATGIQAQGGTNINDAMLMAVQLLEK	3	98.5%
					VQNDIDIYSLTVDSK	2	100.0%
					VTFELVYEELLAR	2	100.0%
					VTIGLLFWDGPGK	2	100.0%
					WKETLYSVMPGLK	3	95.0%
					YIFHSFIER	2	99.9%
					YIFHSFIER	3	95.4%
F1MMG6	F1MMG6_BOVIN	SCARA3	Uncharacterized protein (Fragment)	99.9%	8.91	2	2
					GPAGGKGPKGDPGSLGPPGPQGPQGQPGEPGPVGER	3	99.9%
					VDSLSEDLSLAQAMYDKK	3	99.9%
F1MMK9	F1MMK9_BOVIN	AMBP	Uncharacterized protein	100.0%	23.3	6	6
					EYCGIPGEADEELLR	2	99.9%
					GVCESISGTYEK	2	94.5%
					KADSCQLDYSQGPCLGLFK	3	98.3%
					MTMSTVVLK	2	99.2%
					SYIQLWAFDAVK	2	100.0%
					TVEACNLPIVQGPCR	2	99.9%
Q0VCM5	ITIH1_BOVIN	ITIH1	Inter-alpha-trypsin inhibitor heavy chain H1	100.0%	16.23	10	10
F1MMP5	F1MMP5_BOVIN	ITIH1	Uncharacterized protein	100.0%	16.23	10	10
					AAISGENAGLVR	2	100.0%
					GHMLENHVER	3	87.4%
					GMTDQDGLEPIIDKPLDDYLPLEMVGPR	3	100.0%
					GSLVQASPANLEAAR	2	99.9%
					HKQYYEGSEIMVAGR	3	86.1%
					KLDVEASFLPK	2	88.5%
					LTYEEVLR	2	89.4%
					MAVDAAVDGVVIR	2	99.9%
					TAFISDFAITADENAFTGDIK	2	100.0%
					TMEQFSIHIIVGPR	3	91.3%
F1MNV5	F1MNV5_BOVIN	Bt.4210	Uncharacterized protein	100.0%	22.94	9	10
					ATVQVVAGLK	2	99.7%
					CDLYPVKDFVQPPTR	3	95.1%
					KATVQVVAGLK	2	99.7%
					RPPGFSPFR	2	98.2%
					RPPGFSPFR	3	98.2%
					SGNQFVLYR	2	85.9%
					SPDLEPVLR	2	99.8%
					SVQVMK	1	93.3%
					TWQDCDYKDSAQAATGECTATVAK	3	99.9%
					VYPTVNCQSLGQTSLMK	2	100.0%
F1MNW4	F1MNW4_BOVIN	Bt.23843	Uncharacterized protein	100.0%	24.21	17	20
					AHIAFKPTVAQQR	2	100.0%
					AHIAFKPTVAQQR	3	100.0%
					ALDMEDFK	2	99.3%
					ALDMEDFKTEVSIAPGAK	3	100.0%
					FYNQVSTPLLR	2	99.9%
					HADPDFTK	2	100.0%
					HLEVDVR	2	99.6%
					IQPSGGTNINEALLR	2	100.0%
					NDLVSATK	1	93.3%
					SLAPTAATK	1	95.4%
					SLHVLDTFDGHFDGVPVVSK	2	99.8%
					SLHVLDTFDGHFDGVPVVSK	3	100.0%
					TEDHFSVVDFNHNVR	3	100.0%
					TEDVDQVTVYSYK	2	98.7%
					TILQMSLDHHIVTPLTAMVIENEAGDER	3	100.0%
					VANTVIQTK	1	98.9%
					VGELEVFNGYFVHFFAPENMDPIPK	2	82.3%
					VGELEVFNGYFVHFFAPENMDPIPK	3	100.0%
					VQFELHYQEVK	2	88.5%
					VVNHSPQPQNVVFDVQIPK	3	100.0%
F1MPT4	F1MPT4_BOVIN	SDK2	Uncharacterized protein (Fragment)	99.2%	2.04	2	2
					KIQTLQAPPDMAPANVTLR	2	94.3%
					TGALGHGEMMSLDESSFPALELNNRR	3	85.3%
F1MPT5	F1MPT5_BOVIN	DST	Uncharacterized protein (Fragment)	100.0%	0.57	2	2
					NVSILRAAHEGLIDR	3	85.9%
					TIAEGENLLLKTQGSEKVALQLQLNTIK	3	99.9%
F1MRD0	F1MRD0_BOVIN	Bt.97896	Uncharacterized protein	100.0%	6.93	2	2
					GYSFTTTAER	2	93.1%
					SYELPDGQVITIGNER	2	99.9%
Q3ZC55	ACTN2_BOVIN	ACTN2	Alpha-actinin-2	100.0%	3.8	2	2
F1MRD4	F1MRD4_BOVIN	ACTN2	Uncharacterized protein (Fragment)	100.0%	3.99	2	2
					HLDIPKMLDAEDIVNTPK	3	99.9%
					ISSSNPYSTVTVDEIR	3	99.9%
P41361	ANT3_BOVIN	SERPINC1	Antithrombin-III	100.0%	31.83	11	15
F1MSZ6	F1MSZ6_BOVIN	SERPINC1	Uncharacterized protein	100.0%	31.83	11	15

					AFLEVNEEGSEAAASTVISIAGR	2	100.0%
					AFLEVNEEGSEAAASTVISIAGR	3	100.0%
					ANSHFATAFYQHLADSK	3	84.1%
					DIPVNPMCIYR	2	99.9%
					EQLQDMGLEDLFSPEK	2	100.0%
					EVALNTIIFMGR	2	100.0%
					FRIEDSFSVK	2	95.8%
					FRIEDSFSVK	3	99.4%
					GDDITMVLILPK	2	96.6%
					SDLYVSDAFHK	2	99.9%
					SDLYVSDAFHK	3	99.9%
					SPVEDVCTAKPR	2	100.0%
					SPVEDVCTAKPR	3	98.7%
					TSDQIHFFFAK	3	99.7%
					VAESTQVLELPFKGDDITMVLILPK	3	99.9%
F1MT13	F1MT13_BOVIN	PDZD2	Uncharacterized protein (Fragment)	99.9%	1.93	2	2
					GAGPMAEGAPSTGAGLPQDLMSGEK	3	99.9%
					VQDSSIQVTVTGYRPGGTVEKESLDK	3	99.9%
P35445	COMP_BOVIN	COMP	Cartilage oligomeric matrix protein	99.3%	5.82	3	3
F1MTZ9	F1MTZ9_BOVIN	COMP	Uncharacterized protein	99.3%	5.83	3	3
					DTDLDGFPDEK	2	99.0%
					FYEGPELVADSNVILDTTMR	2	97.3%
					QMEQTYWQANPFR	2	86.1%
F1MU08	F1MU08_BOVIN	Bt.4803	Uncharacterized protein	99.9%	5.51	2	2
P18493	PARP1_BOVIN	PARP1	Poly [ADP-ribose] polymerase 1	99.9%	5.51	2	2
					LEQMPSKEDAIEHFMKLYEEK	3	99.9%
					QDRIFPPESSTPVGAAAPPSAASAPAAVHSGPPDK	3	82.0%
F1MUY2	F1MUY2_BOVIN	KRT6C	Uncharacterized protein	100.0%	2.98	2	2
					FASFIDK	2	82.0%
					NKYEDEINKR	2	81.5%
F1MVK1	F1MVK1_BOVIN		Uncharacterized protein (Fragment)	100.0%	2.91	4	4
					AAGLAFSDGDHR	2	99.8%
					FGLLGEDGEK	2	92.9%
					QTDMQGVNLLFSSR	2	100.0%
					SFFPENWLWK	2	99.4%
F1MXJ6	F1MXJ6_BOVIN	KALRN	Uncharacterized protein (Fragment)	99.9%	1.33	2	2
					LQGFEGTLTAQGK	2	82.1%
					VFLFEQIVIFSELLRKGSLTPGYMFK	3	99.9%
F1MXV8	F1MXV8_BOVIN	SERPINA3-7	Uncharacterized protein	100.0%	21.58	8	9
					DAEAFYASEVLSTNFK	2	100.0%
					DAEAFYASEVLSTNFK	3	97.7%
					DILSQLGIK	2	99.7%
					DILSQLGIKK	2	98.8%
					FSISSHYQLK	2	96.4%
					GTSVDGHSLASSNTDFAFSLYK	3	100.0%
					KIFTSDADFSGITDDHK	2	98.8%
					LAVSHVIHK	1	96.1%
					LINEYVK	2	96.2%
F1MY85	F1MY85_BOVIN	C5	Uncharacterized protein	100.0%	2.44	2	2
					GLLIGEIMSAVLSQEGIDSLTR	2	99.9%
					YVYPLDSSTWIEYWPTDSR	2	100.0%
F1MYN5	F1MYN5_BOVIN	Bt.61392	Uncharacterized protein	100.0%	12.75	6	6
					AGYYFDGISR	2	100.0%
					AITPAYPANHADIIFDITDGNLR	3	100.0%
					GQETADFAPGDGGDLQETAK	2	100.0%
					GYHLNEEGTR	2	98.5%
					VSEIEEEQEDPYLNDR	3	98.0%
					YMDGMTVGVVR	2	100.0%
F1MYZ3	F1MYZ3_BOVIN	Bt.18271	Uncharacterized protein (Fragment)	99.9%	1.06	2	2
					FVLRPHTLNSTSTSKSFQSTVTGELSAPYSK	3	99.9%
					PRLPFPAVPGNVIEAPSHPR	3	99.9%
F1MZN6	F1MZN6_BOVIN	Bt.14715	Uncharacterized protein (Fragment)	100.0%	47.42	15	17
					AIHLTMPQLTLK	2	83.8%
					AIHLTMPQLTLK	3	99.8%
					ASEVGLLLNFMGFHMYK	3	97.7%
					ASYDLQDLLAQAK	2	100.0%
					FMQSVTGWNMGR	2	99.8%
					GFSLLPGLK	2	97.1%
					KVEALTFQHNFLTR	2	100.0%
					LLLSTVVGLFTAPGLHLK	3	100.0%
					LPTLLGAEANLSK	2	100.0%
					LQAFLGVPGEGQGCTSR	2	100.0%
					QPFVQSLSSFAPITLPR	2	100.0%
					SNCDQLEKPSVETPPDPTFTPVPIQTK	3	100.0%
					SSAVDEEALWEQLVR	2	100.0%
					VLNSVLFELK	2	100.0%
					VLSSLQTIQGLLVAQGGASSQAR	2	92.5%
					VLSSLQTIQGLLVAQGGASSQAR	3	100.0%
					VYVHPFHLLVYSK	3	100.0%
F1N1I6	F1N1I6_BOVIN	GSN	Uncharacterized protein	100.0%	17.41	9	10
					AGALNSNDAFVLK	2	99.9%
					AQPVQVAEGSEPDSFWEALGGK	2	100.0%
					HVVPNEVVVQR	2	99.9%
					HVVPNEVVVQR	3	96.1%
					NWRDPDQTDGPGLSYLSSHIANVER	3	99.9%
					PGSMVVEHPEFLK	3	99.7%
					QTQVSVLPEGGETPLFK	2	98.4%
					TGALELLR	2	98.4%
					TPSAAYLWVGAGASEAEK	2	100.0%
					YIETDPANR	2	99.4%
F1N290	F1N290_BOVIN	COBLL1	Uncharacterized protein (Fragment)	99.9%	8.39	2	2
					CLDASSVDDSVESSAFIMDQK	3	99.9%
					SNTISKQYISNTLPSDAPK	3	99.9%
F1N2D3	F1N2D3_BOVIN	ZO1	Uncharacterized protein (Fragment)	100.0%	2.45	2	2
					DESHSTSFKPPEVTSK	3	83.7%
					VAMVNGVSMDNVEHAFAVQQLRKSGK	3	99.9%
F1N2G1	F1N2G1_BOVIN	BOD1L	Uncharacterized protein	100.0%	1.34	2	2
					SQGKQLKASETESQENTTK	3	99.9%
					VGVAVDQVVGMSTKTDTIGLK	3	85.8%
P01030	CO4_BOVIN	Complement C4	Uncharacterized protein (Fragment)	100.0%	20.63	25	28

					AAGLAFSDGDHR	2	99.8%
					AAGLAFSDGDHR	3	95.9%
					AELADQAASWLTR	2	100.0%
					ALVALGAVDTALYAVGGK	3	100.0%
					DGSYGAWLHR	2	98.2%
					DSSTWLTAFVLK	2	99.9%
					EVFAPSSIFQDNFLIPDISAPGTWK	2	99.9%
					FGLLGEDGEK	2	92.9%
					FVLSPFSLDLSK	2	82.4%
					GHLFLQTDQPVYNPGQQVR	3	99.9%
					GSFDFPVGDAISK	2	99.9%
					LEEELQFSLGSK	2	99.9%
					LLLPDSLTTWEIHGVSLSK	3	99.5%
					LQDAPSGQVVR	2	99.9%
					MRPATDILTVTVENSQGFR	3	91.6%
					PVGFSVVPIAAAAVSLK	2	99.6%
					PVGFSVVPIAAAAVSLK	3	97.2%
					QTDMQGVNLLFSSR	2	100.0%
					SFFPENWLWK	2	99.4%
					SHKPLDMVK	3	85.7%
					SHVLQLTNHQVHR	3	100.0%
					TLEIPGNSDPNIIPEGDFK	2	100.0%
					VDYGFQVK	2	98.8%
					VGETLNLNLR	2	99.7%
					VGLSGMAIADITLLSGFHALR	3	100.0%
					VTASDPLEALGSEGALSPGGLASLLR	2	87.9%
					VTASDPLEALGSEGALSPGGLASLLR	3	99.0%
					YVLPNFEVK	2	87.2%
Q28178	TSP1_BOVIN	THBS1	Thrombospondin-1	100.0%	11.71	11	11
F1N3A1	F1N3A1_BOVIN	THBS1	Uncharacterized protein	100.0%	11.71	11	11
					AGTLDLSLTVQGK	2	99.7%
					FQDLVDAVR	2	98.4%
					FVFGTTPEDILR	2	100.0%
					GGVNDNFQGVLQNVR	2	100.0%
					GPDPSSPAFR	2	88.3%
					IEDANLIPPVPDKK	3	99.9%
					MENAELDVPIQSIFTR	2	90.9%
					NALWHTGNTSGQVR	3	84.9%
					QVTQSYWDTNPTR	2	90.3%
					SITLFVQEDR	2	100.0%
					TIVTTLQDSIR	2	97.5%
Q32PJ2	APOA4_BOVIN	APOA4	Apolipoprotein A-IV	100.0%	6.32	2	2
F1N3Q7	F1N3Q7_BOVIN	APOA4	Uncharacterized protein	100.0%	6.32	2	2
					LGEVSTYTDDLQK	2	95.4%
					TQVDTQAQQLR	2	97.0%
F1N4M7	F1N4M7_BOVIN	Bt.64790	Uncharacterized protein	100.0%	7.77	4	5
					DSFQCVNGK	2	87.4%
					EAAEMLTADMDAER	2	99.9%
					GFQLGALDTHR	2	99.7%
					GFQLGALDTHR	3	96.9%
					VANYFDWISQHVGR	3	100.0%
F1N5M2	F1N5M2_BOVIN	Bt.53584	Uncharacterized protein	100.0%	37.97	15	18
					ELSSFIQK	2	98.6%
					FMYEYSINYGQAPLTLLVGYTK	2	100.0%
					FMYEYSINYGQAPLTLLVGYTK	3	100.0%
					FPDATETDLQELVAK	2	100.0%
					GKFPDATETDLQELVAK	2	100.0%
					GKFPDATETDLQELVAK	3	100.0%
					GQELCADYSENTFTEYK	2	100.0%
					HFSLLTIMTNR	2	100.0%
					HQPQEFPTYVEPTNDEICEAFR	3	100.0%
					HQPQEFPTYVEPTNDEICEAFRK	3	100.0%
					ICSQYAAYGK	2	99.9%
					IPEVFLTK	2	99.8%
					LCDNLSTK	2	94.4%
					SYLSMVGSCCTSPNPTVCFLK	2	100.0%
					TRIPEVFLTK	3	99.9%
					VLDQYIFELSR	2	100.0%
					VPTAHLEDVLPLAEDITTILSK	2	100.0%
					VPTAHLEDVLPLAEDITTILSK	3	100.0%
Q3ZBU3	CP059_BOVIN		Uncharacterized protein C16orf59 homolog	99.9%	7.22	2	2
F1N6Y6	F1N6Y6_BOVIN	Bt.49344	Uncharacterized protein	99.9%	7.22	2	2
					ASAPPSTSRCTGSR	3	99.9%
					LPMCASSLWAQLSSMQTR	3	99.9%
F1N757	F1N757_BOVIN		Uncharacterized protein	100.0%	0.19	3	3
					AGPGKPSDASKAVYAQDPLYPPGPPAFPK	3	100.0%
					DVWMPVTSASAKTTCK	3	84.9%
					RCNEHLVPVLTYTAKGLEEGK	3	99.9%
F6RF21	F6RF21_BOVIN	Bt.3161	Uncharacterized protein	99.9%	2	2	2
					EAIYSGFIR	3	99.9%
					PLPVPRSLNSDISYFGVGGKQAVFFVGQSAR	3	99.9%
P02081	HBBF_BOVIN		Hemoglobin fetal subunit beta	100.0%	89.66	15	21
F6RFA2	F6RFA2_BOVIN	Bt.102944	Uncharacterized protein (Fragment)	100.0%	80.25	15	21
					AAVTSLFAK	1	92.5%
					AAVTSLFAK	2	99.9%
					FFESFGDLSSADAILGNPK	2	100.0%
					FFESFGDLSSADAILGNPK	3	100.0%
					FGSEFSPELQASFQK	2	100.0%
					FGSEFSPELQASFQK	3	99.7%
					GAFASLSELHCDK	2	99.0%
					GAFASLSELHCDKLHVDPENFR	3	99.1%
					KVLDSFCEGLK	2	99.4%
					LHVDPENFR	3	99.2%
					LLGNVLVVVLAR	2	100.0%
					LLVVYPWTQR	2	99.6%
					QLDDLKGAFASLSELHCDK	3	92.2%
					RFGSEFSPELQASFQK	2	100.0%
					RFGSEFSPELQASFQK	3	98.4%
					VDEVGGEALGR	2	99.9%
					VKVDEVGGEALGR	2	100.0%

					VKVDEVGGEALGR	3	97.7%
					VLDSFCEGLK	2	99.8%
					VVTGVANALAHR	2	100.0%
					VVTGVANALAHR	3	99.9%
P18902	RET4_BOVIN	RBP4	Retinol-binding protein 4	100.0%	11.48	2	2
G1K122	G1K122_BOVIN	RBP4	Uncharacterized protein	100.0%	10.45	2	2
					DPSGFSPEVQK	2	99.7%
					YWGVASFLQK	2	95.6%
G3MXL3	G3MXL3_BOVIN	LOC100299984	Uncharacterized protein (Fragment)	100.0%	3.9	2	2
					LLRDYQELMNVK	3	99.9%
					SLDLDSIIAEVK	2	99.9%
G3MYZ3	G3MYZ3_BOVIN	Bt.106415	Uncharacterized protein	100.0%	14.57	7	7
					DADQANFMAEFLYEYSR	2	100.0%
					ESFLNNYVYEVSR	2	100.0%
					LAPQLSTEELTFLGK	2	93.7%
					NINPAVDHCCK	2	80.7%
					QILQSINIAILSQK	2	100.0%
					QLTSLLEDVSSK	2	99.9%
					TNFAFR	2	94.1%
G3N022	G3N022_BOVIN	Bt.39893	Uncharacterized protein	99.9%	0.82	2	2
					QRSPASDLELR	3	99.9%
					SREPVPPEDATAEEKR	3	99.9%
G3N0V2	G3N0V2_BOVIN	Bt.96692	Uncharacterized protein	100.0%	12.21	6	6
					DYQELMNTK	2	99.9%
					FLEQQNQVLQTK	2	92.0%
					GGSGGGGGGSSGGSFISSGGRASSTK	3	86.6%
					LLRDYQELMNTK	3	87.8%
					NTKVEISELNR	3	89.9%
					SLNNQFASFIDKVR	2	99.9%
G3N1S7	G3N1S7_BOVIN	Bt.31944	Uncharacterized protein (Fragment)	99.9%	3.25	2	2
					MEETVSQLMISK	3	99.9%
					VINLEALQTLSVNTTLEELK	3	99.9%
G3N1U4	G3N1U4_BOVIN	Bt.92049	Uncharacterized protein	100.0%	18.98	7	9
					DTQSIIFLGK	2	99.9%
					RIHELYLPK	2	99.8%
					RIHELYLPK	3	100.0%
					RVDGHTLASSNTDFAFSLYK	3	100.0%
					SLINDYVK	2	94.6%
					VDGHTLASSNTDFAFSLYK	2	100.0%
					VDGHTLASSNTDFAFSLYK	3	100.0%
					VLYSSEAFPTNFGDPEAAK	2	100.0%
					VNRPFLIAVVLK	3	100.0%
G3N2D0	G3N2D0_BOVIN	ZNF469	Uncharacterized protein	100.0%	1.6	2	2
					SLEDLPACWEGGGVVTHFLSGIAGQASR	3	99.9%
					TPESLQPEDLRPLNAEAPPSQGTRAGLR	3	99.9%
G3X6N3	G3X6N3_BOVIN	Bt.5336	Uncharacterized protein	100.0%	66.05	47	66
					AAANFFSASCVPCADQSSFPK	2	100.0%
					AAANFFSASCVPCADQSSFPK	3	94.3%
					CACSNHEPYFGYSGAFK	2	100.0%
					CACSNHEPYFGYSGAFK	3	100.0%
					CGLVPVLAENYK	2	100.0%
					CLMEGAGDVAFVK	2	100.0%
					DGTRKPVTDAENCHLAR	3	99.6%
					DKPDNFQLFQSPHGK	2	100.0%
					DKPDNFQLFQSPHGK	3	100.0%
					DLLFRDDTK	2	98.4%
					DNPQTHYYAVAVVK	2	100.0%
					DNPQTHYYAVAVVK	3	96.1%
					DQTVIQNTDGNNNEAWAK	2	100.0%
					DSADGFLK	1	98.6%
					DSADGFLK	2	98.4%
					DTDFK	1	84.6%
					DTDFKLNELR	2	98.7%
					EDVIWELLNHAQEHFGK	3	99.9%
					ELPDPQESIQR	2	99.5%
					ENFEVLCK	1	100.0%
					ESKPPDSSKDECMVK	2	99.8%
					FDEFFSAGCAPGSPR	2	100.0%
					GDVAFVK	1	91.2%
					GDVAFVK	2	93.4%
					GDVAFVKDQTVIQNTDGNNNEAWAK	3	100.0%
					GEADAMSLDGGYLYIAGK	2	100.0%
					GYLAVAVVK	1	94.9%
					GYLAVAVVK	2	100.0%
					HSTVFDNLPNPEDR	2	100.0%
					HSTVFDNLPNPEDR	3	99.8%
					HSTVFDNLPNPEDRK	2	100.0%
					HSTVFDNLPNPEDRK	3	100.0%
					ILESGPFVSCVK	2	100.0%
					IMKGEADAMSLDGGYLYIAGK	2	100.0%
					IMKGEADAMSLDGGYLYIAGK	3	100.0%
					KENFEVLCK	2	100.0%
					KENFEVLCK	3	89.2%
					KNYELLCGDNTR	2	100.0%
					KNYELLCGDNTRK	3	98.9%
					KPVTDAENCHLAR	2	100.0%
					KPVTDAENCHLAR	3	100.0%
					KTYDSYLGDDYVR	2	100.0%
					KTYDSYLGDDYVR	3	100.0%
					LCQLCAGK	1	92.9%
					LYKELPDPQESIQR	2	99.8%
					LYKELPDPQESIQR	3	100.0%
					MDFELYLGYEYVTALQNLR	2	100.0%
					NYELLCGDNTR	2	100.0%
					NYELLCGDNTRK	2	99.7%
					QQDDFGK	1	95.9%
					QQDDFGK	2	93.0%
					SAGWNIPMGK	2	100.0%
					SVTDCTSNFCLFQSNSK	2	100.0%
					TAGWNIPMGLLYSK	2	100.0%

					TSDANINWNNLK	1	97.4%
					TSDANINWNNLK	2	100.0%
					TSHMDCIK	2	99.9%
					TVGGKEDVIWELLNHAQEHFGK	2	100.0%
					TVGGKEDVIWELLNHAQEHFGK	3	100.0%
					TYDSYLGDDYVR	1	96.8%
					TYDSYLGDDYVR	2	100.0%
					WCAIGHQER	2	99.8%
					WCTISTHEANK	3	99.3%
					WSGFSGGAIECETAENTEECIAK	2	100.0%
					YYGYTGAFR	1	100.0%
					YYGYTGAFR	2	100.0%
G3X7A5	G3X7A5_BOVIN	Bt.19562	Uncharacterized protein	100.0%	39.07	49	56
					AAVYNHFISDGVK	2	100.0%
					ACEPGVDYVYK	2	99.1%
					AGDFLENHYR	2	99.4%
					AGDFLENHYR	3	98.1%
					AGQYSSDLRK	2	100.0%
					AQFILQGDACVK	2	99.9%
					AYYEDSPQQVFSAEFEVK	2	100.0%
					DICEAQVNSLGR	2	99.9%
					DPLTITVR	2	99.3%
					DSITTWEILAVSLSDKK	2	97.0%
					EEVPAADLSDQVPDTESETK	2	100.0%
					EVTLEDRLDK	2	95.4%
					EVVADSVWVDVK	2	99.4%
					EYVLPSFEVQLEPEEK	2	99.9%
					FFKPAMPFDLMVYVTNPDGSPAR	2	99.2%
					FFKPAMPFDLMVYVTNPDGSPAR	3	100.0%
					FVTVVATFGNVQVEK	2	100.0%
					FYHPDKEDGMLSK	2	94.0%
					FYYIDDPDGLK	2	100.0%
					FYYIDDPDGLKVNIIAR	3	99.8%
					GQGTLSVVTVYHAK	3	100.0%
					GSMILDICTK	2	99.1%
					GYTQQLAFR	2	99.2%
					HIPVVTQGSNVQSLTQDDGVAK	2	100.0%
					HIPVVTQGSNVQSLTQDDGVAK	3	100.0%
					HQQTITIPAR	2	100.0%
					IGLHEVEVK	2	95.4%
					ILLQGTPVAQMTEDAIDGER	2	99.7%
					ILLQGTPVAQMTEDAIDGER	3	99.8%
					ISLTHSLTR	2	98.0%
					KDYDTTPPVVR	2	100.0%
					KGYTQQLAFR	3	91.2%
					KQVLSNENTQLNSNNGYLSTVTIK	3	100.0%
					LPYSVVR	2	81.3%
					LSINTQNKR	2	99.0%
					NTLIIYLDK	2	84.6%
					NYAGVFTDAGLTLK	2	100.0%
					QVLLNGVQPSR	2	95.7%
					QVLSNENTQLNSNNGYLSTVTIK	2	100.0%
					QVLSNENTQLNSNNGYLSTVTIK	3	100.0%
					RQESLELIR	2	99.6%
					SDLDDDIIPEEDIISR	2	100.0%
					SSVAVPYVIVPLK	2	98.7%
					TIYTPGSTVLYR	2	98.1%
					TMQALPYNTQGNSNNYLHLSVPR	3	99.7%
					VELKPGETLNVNFHLR	3	100.0%
					VELLYNPAFCSLATAK	2	100.0%
					VHQYFNVGLIQPGAVK	3	92.8%
					VPINDGNGEAILKR	2	98.1%
					VPINDGNGEAILKR	3	100.0%
					VSHTVEDCLSFK	3	99.9%
					VSIRPAPETVK	2	99.6%
					VSIRPAPETVK	3	100.0%
					VYSYYNLDETCIR	2	100.0%
					YLGDQDATMSILDISMMTGFSPDVEDLK	2	100.0%
					YYGGGYGSTQATFMVFQALAQYQK	3	100.0%
G5E5D5	G5E5D5_BOVIN	Bt.72941	Uncharacterized protein	100.0%	0.34	2	2
					PFSPSPPHQERK	3	99.9%
					SLPPLQRPDKK	3	99.9%
O18920	ANGP1_BOVIN	ANGPT1	Angiopoietin-1	99.9%	5.03	2	3
					LTDVETQVLNQTSR	3	99.9%
					QLLQQTNEILK	1	82.4%
					QLLQQTNEILK	3	80.5%
O46375	TTHY_BOVIN	TTR	Transthyretin	100.0%	70.75	9	12
					AADETWEPFASGK	2	100.0%
					CPLMVK	1	90.3%
					GSPAANVGVK	1	96.8%
					HYTIAALLSPYSYSTTALVSSPK	2	100.0%
					HYTIAALLSPYSYSTTALVSSPK	3	100.0%
					KAADETWEPFASGK	2	100.0%
					SLGISPFHEFAEVVFTANDSGPR	2	100.0%
					SLGISPFHEFAEVVFTANDSGPR	3	100.0%
					TSESGELHGLTTEDK	2	100.0%
					TSESGELHGLTTEDKFVEGLYK	2	100.0%
					TSESGELHGLTTEDKFVEGLYK	3	100.0%
					VELDTK	1	97.3%
P00735	THRB_BOVIN	F2	Prothrombin	100.0%	16.16	7	8
					DQDFNPAVPLAENFCR	2	90.9%
					LGEDPDPDAAIEGR	2	99.6%
					NPDGSITGPWCYTTSPTLR	2	98.7%
					SGIECQLWR	2	98.0%
					SPQELLCGASLISDR	2	99.5%
					TSEDHFQPFFNEK	2	99.9%
					TSEDHFQPFFNEK	3	84.4%
					WYQMGIVSWGEGCDR	2	99.2%
P00761	TRYP_PIG		Trypsin	100.0%	14.29	3	4
					LSSPATLNSR	2	99.6%

					VATVSLPR	1	84.0%
					VATVSLPR	2	96.5%
					VCNYVNWIQQTIAAN	2	100.0%
P00978	AMBP_BOVIN	AMBP	Protein AMBP	100.0%	20.74	5	5
					EYCGIPGEADEELLR	2	99.9%
					GVCESISGTYEK	2	94.5%
					KADSCQLDYSQGPCLGLFK	3	98.3%
					SYIQLWAFDAVK	2	100.0%
					TVEACNLPIVQGPCR	2	99.9%
P01035	CYTC_BOVIN	CST3	Cystatin-C	100.0%	31.08	2	2
					KQVVSGMNYFLDVELGR	3	96.6%
					LLGGLMEADVNEEGVQEALSFAVSEFNKR	3	99.8%
P01044	KNG1_BOVIN	KNG1	Kininogen-1	100.0%	7.41	5	5
					ATVQVVAGLK	2	99.7%
					KATVQVVAGLK	2	99.7%
					RPPGFSPFR	3	98.2%
					SPDLEPVLR	2	99.8%
					VYPTVNCQPLGQTSLMK	2	100.0%
P01044-2	KNG1_BOVIN	KNG1	Isoform LMW of Kininogen-1	100.0%	8.49	4	4
					ATVQVVAGLK	2	99.7%
					KATVQVVAGLK	2	99.7%
					SPDLEPVLR	2	99.8%
					VYPTVNCQPLGQTSLMK	2	100.0%
P01045	KNG2_BOVIN	KNG2	Kininogen-2	100.0%	13.57	8	10
					ATVQVVGGLK	2	99.1%
					LISDFPETTSPK	2	97.8%
					LNAEHDGTFYFK	2	97.1%
					LNAEHDGTFYFK	3	98.8%
					RPPGFSPFR	2	98.2%
					RPPGFSPFR	3	98.2%
					SGNQFVLYR	2	85.9%
					SPDLEPVLR	2	99.8%
					SVQVMK	1	93.3%
					VYPTVNCQPLGQTSLMK	2	100.0%
P01045-2	KNG2_BOVIN	KNG2	Isoform LMW of Kininogen-2	100.0%	14.52	6	8
					ATVQVVGGLK	2	99.1%
					LNAEHDGTFYFK	2	97.1%
					LNAEHDGTFYFK	3	98.8%
					RPPGFSPFR	2	98.2%
					RPPGFSPFR	3	98.2%
					SPDLEPVLR	2	99.8%
					SVQVMK	1	93.3%
					VYPTVNCQPLGQTSLMK	2	100.0%
P01966	HBA_BOVIN	HBA	Hemoglobin subunit alpha	100.0%	58.45	6	10
					AVEHLDDLPGALSELSDLHAHK	2	99.6%
					AVEHLDDLPGALSELSDLHAHK	3	100.0%
					FLANVSTVLTSK	2	100.0%
					LRVDPVNFK	2	80.9%
					LRVDPVNFK	3	97.1%
					MFLSFPTTK	2	99.9%
					TYFPHFDLSHGSAQVK	2	100.0%
					TYFPHFDLSHGSAQVK	3	100.0%
					VGGHAAEYGAEALER	2	100.0%
					VGGHAAEYGAEALER	3	100.0%
P02070	HBB_BOVIN	HBB	Hemoglobin subunit beta	100.0%	23.45	3	3
					EFTPVLQADFQK	2	99.6%
					LLGNVLVVVLAR	2	100.0%
					LLVVYPWTQR	2	99.6%
P02769	ALBU_BOVIN	ALB	Serum albumin	100.0%	77.76	59	102
					AEFVEVTK	1	98.7%
					AEFVEVTK	2	99.6%
					AEFVEVTKLVTDLTK	2	99.9%
					AEFVEVTKLVTDLTK	3	100.0%
					AFDEKLFTFHADICTLPDTEK	3	99.8%
					ATEEQLK	1	83.7%
					ATEEQLKTVMENFVAFVDK	2	100.0%
					ATEEQLKTVMENFVAFVDK	3	100.0%
					CCAADDKEACFAVEGPK	2	100.0%
					CCAADDKEACFAVEGPK	3	100.0%
					CCTESLVNR	1	98.8%
					CCTESLVNR	2	100.0%
					DAFLGSFLYEYSR	1	100.0%
					DAFLGSFLYEYSR	2	100.0%
					DAFLGSFLYEYSR	3	99.9%
					DAIPENLPPLTADFAEDK	2	95.5%
					DAIPENLPPLTADFAEDKDVCK	2	100.0%
					DDPHACYSTVFDK	1	100.0%
					DDPHACYSTVFDK	2	100.0%
					DDPHACYSTVFDK	3	100.0%
					DDPHACYSTVFDKLK	2	100.0%
					DDPHACYSTVFDKLK	3	100.0%
					DLGEEHFK	1	98.4%
					DLGEEHFK	2	99.7%
					EACFAVEGPK	2	98.1%
					ECCHGDLLECADDR	2	99.9%
					ECCHGDLLECADDR	3	87.7%
					ECCHGDLLECADDRADLAK	2	99.3%
					ECCHGDLLECADDRADLAK	3	99.9%
					ETYGDMADCCEK	2	100.0%
					EYEATLEECCAK	2	100.0%
					FKDLGEEHFK	1	100.0%
					FKDLGEEHFK	2	99.5%
					FKDLGEEHFK	3	100.0%
					FPKAEFVEVTK	3	99.1%
					GLVLIAFSQYLQQCPFDEHVK	2	100.0%
					GLVLIAFSQYLQQCPFDEHVK	3	100.0%
					HLVDEPQNLIK	1	100.0%
					HLVDEPQNLIK	2	100.0%
					HLVDEPQNLIK	3	99.8%
					HLVDEPQNLIKQNCDQFEK	3	99.6%

					HPEYAVSVLLR	2	99.9%
					HPYFYAPELLYYANK	2	100.0%
					HPYFYAPELLYYANK	3	100.0%
					KQTALVELLK	1	98.9%
					KQTALVELLK	2	99.9%
					KQTALVELLK	3	100.0%
					KVPQVSTPTLVEVSR	1	100.0%
					KVPQVSTPTLVEVSR	2	100.0%
					KVPQVSTPTLVEVSR	3	100.0%
					LCVLHEK	2	99.3%
					LCVLHEKTPVSEKVTK	3	100.0%
					LFTFHADICTLPDTEK	2	100.0%
					LFTFHADICTLPDTEK	3	100.0%
					LGEYGFQNALIVR	1	100.0%
					LGEYGFQNALIVR	2	100.0%
					LGEYGFQNALIVR	3	100.0%
					LKECCDKPLLEK	2	99.9%
					LKECCDKPLLEK	3	99.9%
					LKHLVDEPQNLIK	2	98.8%
					LKHLVDEPQNLIK	3	100.0%
					LKPDPNTLCDEFK	2	100.0%
					LKPDPNTLCDEFK	3	100.0%
					LKPDPNTLCDEFKADEK	2	99.9%
					LKPDPNTLCDEFKADEK	3	100.0%
					LVNELTEFAK	2	100.0%
					LVTDLTK	1	90.9%
					LVTDLTK	2	90.0%
					LVVSTQTALA	2	99.9%
					MPCTEDYLSLILNR	2	100.0%
					MPCTEDYLSLILNR	3	99.9%
					NECFLSHKDDSPDLPK	2	100.0%
					NYQEAKDAFLGSFLYEYSR	2	99.7%
					NYQEAKDAFLGSFLYEYSR	3	99.3%
					PCFSALTPDETYVPK	2	99.8%
					QEPERNECFLSHKDDSPDLPK	2	99.6%
					QEPERNECFLSHKDDSPDLPK	3	100.0%
					QNCDQFEK	2	96.0%
					QNCDQFEKLGEYGFQNALIVR	3	100.0%
					QTALVELLK	2	91.3%
					RHPEYAVSVLLR	2	100.0%
					RHPEYAVSVLLR	3	100.0%
					RHPYFYAPELLYYANK	2	100.0%
					RHPYFYAPELLYYANK	3	100.0%
					RPCFSALTPDETYVPK	2	99.8%
					RPCFSALTPDETYVPK	3	98.7%
					SHCIAEVEK	1	100.0%
					SHCIAEVEK	2	85.5%
					SHCIAEVEKDAIPENLPPLTADFAEDKDVCK	3	100.0%
					SLHTLFGDELCK	1	100.0%
					SLHTLFGDELCK	2	100.0%
					SLHTLFGDELCK	3	100.0%
					TVMENFVAFVDK	1	100.0%
					TVMENFVAFVDK	2	100.0%
					TVMENFVAFVDK	3	99.9%
					VHKECCHGDLLECADDRADLAK	3	95.7%
					VPQVSTPTLVEVSR	2	100.0%
					VTKCCTESLVNR	3	93.3%
					YICDNQDTISSK	1	100.0%
					YICDNQDTISSK	2	100.0%
					YNGVFQECCQAEDK	2	100.0%
					YNGVFQECCQAEDKGACLLPK	3	100.0%
P07224	PROS_BOVIN	PROS1	Vitamin K-dependent protein S	100.0%	7.7	4	4
					GWNLMNQGTSGVK	2	99.9%
					VINDGLWHMVSVEELEQSISVK	3	99.9%
					VYFAGVPR	2	94.3%
					YLGCLGSFR	2	92.4%
P12763	FETUA_BOVIN	AHSG	Alpha-2-HS-glycoprotein	100.0%	55.99	17	27
					AQFVPLPVSVSVEFAVAATDCIAK	2	100.0%
					AQFVPLPVSVSVEFAVAATDCIAK	3	100.0%
					CDSSPDSAEDVR	1	88.2%
					CDSSPDSAEDVR	2	99.9%
					CNLLAEK	1	98.1%
					EPACDDPDTEQAALAAVDYINK	2	100.0%
					EPACDDPDTEQAALAAVDYINK	3	100.0%
					EVVDPTKCNLLAEK	2	100.0%
					EVVDPTKCNLLAEK	3	81.0%
					EVVDPTKCNLLAEKQYGFCK	3	98.9%
					GSVIQKALGGEDVR	2	98.5%
					GSVIQKALGGEDVR	3	99.8%
					GYKHTLNQIDSVK	2	100.0%
					GYKHTLNQIDSVK	3	100.0%
					GYKHTLNQIDSVKVWPR	3	100.0%
					HTFSGVASVESSSGEAFHVGK	2	100.0%
					HTLNQIDSVK	1	100.0%
					HTLNQIDSVKVWPR	3	99.0%
					QDGQFSVLFTK	1	97.9%
					QDGQFSVLFTK	2	100.0%
					QQTQHAVEGDCDIHVLK	2	100.0%
					QQTQHAVEGDCDIHVLK	3	100.0%
					QYGFCK	1	96.0%
					QYGFCK	2	92.8%
					TPIVGQPSIPGGPVR	1	100.0%
					TPIVGQPSIPGGPVR	2	100.0%
					VVHAVEVALATFNAESNGSYLQLVEISR	3	100.0%
P15497	APOA1_BOVIN	APOA1	Apolipoprotein A-I	100.0%	67.17	24	32
					AKPVLEDLR	2	99.3%
					DFATVYVEAIK	2	100.0%
					DFATVYVEAIKDSGR	3	99.9%
					DSGRDYVAQFEASALGK	2	100.0%
					DSGRDYVAQFEASALGK	3	99.9%

					DYVAQFEASALGK	2	100.0%
					EQLGPVTQEFWDNLEK	2	100.0%
					EQLGPVTQEFWDNLEKETASLR	2	99.9%
					EQLGPVTQEFWDNLEKETASLR	3	100.0%
					KWHEEVEIYR	2	93.3%
					KWHEEVEIYR	3	99.9%
					LEALKEGGGSLAEYHAK	3	100.0%
					LLDNWDTLASTLSK	2	100.0%
					LLDNWDTLASTLSK	3	99.1%
					LSPLAQELR	2	99.6%
					QEMHKDLEEVK	2	98.9%
					QGLLPVLESLK	2	100.0%
					QQLAPYSDDLR	2	100.0%
					VAPLGEEFR	1	96.3%
					VAPLGEEFR	2	99.1%
					VKDFATVYYEAIK	2	98.8%
					VKDFATVYYEAIK	3	100.0%
					VKDFATVYYEAIKDSGR	3	100.0%
					VQPYLDEFQK	2	98.0%
					VQPYLDEFQKK	2	80.7%
					VREQLGPVTQEFWDNLEK	2	100.0%
					VREQLGPVTQEFWDNLEK	3	100.0%
					VREQLGPVTQEFWDNLEKETASLR	3	100.0%
					VSILAAIDEASK	2	100.0%
					VSILAAIDEASKK	2	99.9%
					WHEEVEIYR	2	99.9%
					WHEEVEIYR	3	99.2%
P17690	APOH_BOVIN	APOH	Beta-2-glycoprotein 1	100.0%	26.38	7	7
					ATVIYEGER	2	90.0%
					EHSSLAFWK	3	97.4%
					FTCPLTGLWPINTLK	2	100.0%
					RATVIYEGER	2	99.5%
					TCPKPDELPFSTVVPLKR	3	100.0%
					TYEPGEQIVFSCQPGYVSR	2	99.9%
					WSPDLPVCAPITCPPPPIPK	2	86.8%
P17697	CLUS_BOVIN	CLU	Clusterin	100.0%	11.85	5	5
					ASSIMDELFQDR	2	99.8%
					KLLLSSLEEAK	3	99.4%
					KLLLSSLEEAKK	3	99.5%
					LYDQLLQSYQQK	2	99.8%
					TPYHFPTMEFTENNDR	3	92.4%
P19035	APOC3_BOVIN	APOC3	Apolipoprotein C-III	100.0%	28.13	2	3
					DALSSVQESQVAQQAR	2	100.0%
					DALSSVQESQVAQQAR	3	100.0%
					DWMTESFSSLK	2	99.7%
P28800	A2AP_BOVIN	SERPINF2	Alpha-2-antiplasmin	100.0%	27.64	9	11
					GEDLANINR	1	96.1%
					GEDLANINR	2	98.9%
					GFPIKEDFLEQSEQLFGAK	2	100.0%
					LCQDLGPGAFR	2	100.0%
					LDNQEPGGQIAPK	2	100.0%
					LGPPSEEDYAQPSSPK	2	100.0%
					LKEVLHADSGPCLPHLLSR	3	99.9%
					LVVSSVQHQSALELSEAGVQAAAATSTAMSR	3	98.8%
					PMSLTGMKGEDLANINR	3	95.9%
					SKFDPNLTQR	2	99.1%
					SKFDPNLTQR	3	97.3%
P34955	A1AT_BOVIN	SERPINA1	Alpha-1-antiproteinase	100.0%	50.24	27	44
					AALTIDEK	1	98.7%
					AALTIDEK	2	99.7%
					ADLSGITK	2	99.7%
					DFHVDEQTTVK	1	100.0%
					DFHVDEQTTVK	2	99.9%
					DFHVDEQTTVK	3	99.8%
					DFHVDEQTTVKVPMMNR	3	99.5%
					GKWEKPFEMK	2	99.5%
					GKWEKPFEMK	3	93.8%
					GNTHTEILK	1	97.7%
					GSHGKIVELVK	3	94.9%
					HTTERDFHVDEQTTVK	2	100.0%
					HTTERDFHVDEQTTVK	3	100.0%
					INDYVEK	1	90.0%
					KINDYVEK	1	98.7%
					KINDYVEK	2	99.9%
					KYASSANLHLPK	2	100.0%
					KYASSANLHLPK	3	100.0%
					LGMFDLHYCDK	1	100.0%
					LGMFDLHYCDK	2	100.0%
					LGMFDLHYCDK	3	99.9%
					LNNELLAK	2	97.2%
					LQQLEDKLNNELLAK	2	100.0%
					LQQLEDKLNNELLAK	3	99.9%
					LQQLEDKLNNELLAKFLEK	3	96.9%
					LSISETYDLK	1	97.3%
					LSISETYDLK	2	100.0%
					LVDTFLEDVK	1	100.0%
					LVDTFLEDVK	2	100.0%
					NLYHSEAFSINFR	2	99.9%
					NLYHSEAFSINFR	3	100.0%
					NTKSPLFVGK	3	98.9%
					PFLCILYDR	2	99.4%
					SPLFVGK	1	97.9%
					SPLFVGK	2	99.3%
					SVLGDVGITEVFSDR	1	98.3%
					SVLGDVGITEVFSDR	2	100.0%
					SVLGDVGITEVFSDR	3	100.0%
					SVLGDVGITEVFSDRADLSGITK	3	100.0%
					VLDPNTVFALVNYISFK	3	100.0%
					VVNPTQA	1	98.5%
					WEKPFEMK	2	98.8%

					YASSANLHLPK	2	100.0%
					YASSANLHLPK	3	100.0%
P43481	KIT_BOVIN	KIT	Mast/stem cell growth factor receptor Kit	99.9%	3.79	2	2
					INSVGSSASSTQPLLVHEDV	3	99.9%
					QEDHAEVALYKNLLHSK	3	99.9%
P50448	F12AI_BOVIN		Factor XIIa inhibitor	100.0%	26.92	9	11
					AFMSEGFTSFSQIFHSSDLTIK	3	99.8%
					FHPTHLTMPR	3	99.9%
					FPVFMGR	2	91.4%
					HHLQDLEQALSTAVFK	2	98.9%
					HHLQDLEQALSTAVFK	3	97.3%
					HQATLELTESGVDATAASVVSVAR	3	99.8%
					LILLNAVALSAK	2	100.0%
					LLDSLPEDTR	2	99.8%
					LYQDFSVLK	2	98.6%
					SAEAVLGEALTDFSLR	2	100.0%
					SAEAVLGEALTDFSLR	3	100.0%
P56652	ITIH3_BOVIN	ITIH3	Inter-alpha-trypsin inhibitor heavy chain H3	100.0%	19.64	14	14
					AVDGIEVYSTK	2	99.9%
					DSLVPATPENIQEASK	2	99.9%
					DYIFGDYIER	2	92.9%
					FAHNVVTTR	1	100.0%
					FTVSVNVAAGSK	2	99.8%
					FVMDIQDR	2	94.5%
					GDFIITYDVNR	2	100.0%
					GMTNINDALLR	2	98.6%
					KGHVSFKPSLDQQR	3	84.4%
					LADEDMNSFK	2	99.4%
					LGIASAQMDFR	2	99.2%
					LIQDPVTGLTVNGQIIGEK	3	93.9%
					NAQGEEKEILTAQALELSLK	2	100.0%
					VTFELTYEELLKR	3	99.4%
P60712	ACTB_BOVIN	ACTB	Actin, cytoplasmic 1	100.0%	12.53	3	3
P63258	ACTG_BOVIN	ACTG1	Actin, cytoplasmic 2	100.0%	12.53	3	3
					DLYANTVLSGGTTMYPGIADR	2	99.9%
					GYSFTTTAER	2	93.1%
					SYELPDGQVITIGNER	2	99.9%
P81187	CFAB_BOVIN	CFB	Complement factor B	100.0%	19.71	12	14
					AGQVLEYLCPSGFYPYPTQIR	2	100.0%
					ALLEVYNMMSR	2	99.2%
					DIEALFVSESKK	2	99.2%
					GIPEFYDYDVALVR	2	100.0%
					LQGMENLEDVFVQMLDESR	2	100.0%
					LQGMENLEDVFVQMLDESR	3	100.0%
					RPQQVPGYAR	2	99.9%
					SSEADWVTDQLNQINYADHK	2	96.1%
					SSEADWVTDQLNQINYADHK	3	100.0%
					STGSWSTLQTQDR	2	100.0%
					STGSWSTLQTQDRK	2	99.6%
					VKDVSEVVTPR	2	99.2%
					YGLVTYATEPK	2	100.0%
					YLLDIGR	2	99.2%
P81644	APOA2_BOVIN	APOA2	Apolipoprotein A-II	100.0%	50	5	8
					AGTDLLNFLSSFIDPK	2	100.0%
					KAGTDLLNFLSSFIDPK	2	98.8%
					KAGTDLLNFLSSFIDPK	3	100.0%
					QAEESNLQSLVSQYFQTVADYGK	3	99.3%
					TQEELTPFFK	1	100.0%
					TQEELTPFFK	2	99.9%
					TQEELTPFFKK	2	99.8%
					TQEELTPFFKK	3	100.0%
Q03247	APOE_BOVIN	APOE	Apolipoprotein E	100.0%	18.99	4	4
					AYKEELEGQLGPMAQETQAR	3	100.0%
					FGPLVEQGQSR	2	99.9%
					LAVYQAGASEGAER	2	99.5%
					SEVQAMLGQSTEELR	2	100.0%
Q05443	LUM_BOVIN	LUM	Lumican	100.0%	17.84	5	6
					FSALQYLR	2	99.9%
					ISNIPDEYFK	2	98.9%
					LPSGLPVSLLTLYLDNNK	2	100.0%
					LPSGLPVSLLTLYLDNNK	3	95.5%
					SLEYLDLSFNQMTK	2	100.0%
					SLVDLQLTNNK	2	100.0%
Q17QL7	Q17QL7_BOVIN	KRT15	KRT15 protein	100.0%	6.18	3	3
					ALEEANADLEVK	2	99.9%
					LAADDFR	2	96.1%
					VLDELTLTK	2	99.8%
Q28085	CFAH_BOVIN	CFH	Complement factor H	100.0%	3.64	4	4
					EAFTMIGPR	2	98.6%
					ENYLIQDAEEIVCK	2	97.2%
					NGEWSQLPK	2	87.2%
					TPVILNGQAVLPK	2	99.2%
Q29443	TRFE_BOVIN	TF	Serotransferrin	100.0%	68.47	49	65
					AAANFFSASCVPCADQSSFPK	2	100.0%
					AAANFFSASCVPCADQSSFPK	3	94.3%
					CACSNHEPYFGYSGAFK	2	100.0%
					CACSNHEPYFGYSGAFK	3	100.0%
					CGLVPVLAENYK	2	100.0%
					CLMEGAGDVAFVK	2	100.0%
					DGTRKPVTDAENCHLAR	3	99.6%
					DKPDNFQLFQSPHGK	2	100.0%
					DKPDNFQLFQSPHGK	3	100.0%
					DLLFRDDTK	2	98.4%
					DNPQTHYYAVAVVK	2	100.0%
					DQTVIQNTDGNNNEAWAK	2	100.0%
					DSADGFLK	1	98.6%
					DSADGFLK	2	98.4%
					DTDFK	1	84.6%
					DTDFKLNELR	2	98.7%
					EDVIWELLNHAQEHFGK	3	99.9%

					ELPDPQESIQR	2	99.5%
					ENFEVLCK	1	100.0%
					ESKPPDSSKDECMVK	2	99.8%
					FDEFFSAGCAPGSPR	2	100.0%
					GDVAFVK	2	93.4%
					GDVAFVKDQTVIQNTDGNNNEAWAK	3	100.0%
					GEADAMSLDGGYLYIAGK	2	100.0%
					GYLAVAVVK	1	94.9%
					GYLAVAVVK	2	100.0%
					HSTVFDNLPNPEDR	2	100.0%
					HSTVFDNLPNPEDR	3	99.8%
					HSTVFDNLPNPEDRK	2	100.0%
					HSTVFDNLPNPEDRK	3	100.0%
					ILESGPFVSCVK	2	100.0%
					ILNKQQDDFGK	2	81.8%
					IMKGEADAMSLDGGYLYIAGK	2	100.0%
					IMKGEADAMSLDGGYLYIAGK	3	100.0%
					KENFEVLCK	2	100.0%
					KENFEVLCK	3	89.2%
					KNYELLCGDNTR	2	100.0%
					KNYELLCGDNTRK	3	98.9%
					KPVTDAENCHLAR	2	100.0%
					KPVTDAENCHLAR	3	100.0%
					KTYDSYLGDDYVR	2	100.0%
					KTYDSYLGDDYVR	3	100.0%
					LCQLCAGK	1	92.9%
					LYKELPDPQESIQR	2	99.8%
					LYKELPDPQESIQR	3	100.0%
					MDFELYLGYEYVTALQNLR	2	100.0%
					NYELLCGDNTR	2	100.0%
					NYELLCGDNTRK	2	99.7%
					QQDDFGK	2	93.0%
					SVDDYQECYLAMVPSHAVVAR	2	100.0%
					SVTDCTSNFCLFQSNSK	2	100.0%
					TAGWNIPMGLLYSK	2	100.0%
					TSDANINWNNLK	1	97.4%
					TSDANINWNNLK	2	100.0%
					TSDANINWNNLKDK	2	100.0%
					TSDANINWNNLKDK	3	94.8%
					TSHMDCIK	2	99.9%
					TVGGKEDVIWELLNHAQEHFGK	2	100.0%
					TVGGKEDVIWELLNHAQEHFGK	3	100.0%
					TYDSYLGDDYVR	2	100.0%
					WCAIGHQER	2	99.8%
					WCTISTHEANK	3	99.3%
					WSGFSGGAIECETAENTEECIAK	2	100.0%
					YYGYTGAFR	1	100.0%
					YYGYTGAFR	2	100.0%
Q29RZ2	PPWD1_BOVIN	PPWD1	Peptidylprolyl isomerase domain and WD repeat-con	99.9%	8.07	2	2
					SYMHRDVITHVVCTKTDFIITASHDGHVK	3	99.9%
					TELSAREPVVAVPQENEEEENEER	3	99.9%
Q2KIF2	Q2KIF2_BOVIN	LRG1	Leucine-rich alpha-2-glycoprotein 1	100.0%	20.23	5	6
					FLEASWLHGLK	3	97.0%
					FLLPVPQLK	2	90.5%
					LDMLDLSNNLLTTVPTGLWTSLGK	2	100.0%
					LDMLDLSNNLLTTVPTGLWTSLGK	3	95.4%
					LQVLGEGLLAPQPK	2	99.9%
					NSLTGLFPGFFR	2	100.0%
Q2KIS7	TETN_BOVIN	CLEC3B	Tetranectin	100.0%	13.86	2	2
					NWETEITAQPDGGK	2	98.9%
					TQLDSLAQEVALLK	2	98.8%
Q2KIU3	HP252_BOVIN		Protein HP-25 homolog 2	100.0%	13.02	2	2
					FGFDIELFQHAVK	3	99.9%
					MNDPLPAPSQPIVFK	2	99.8%
Q2KJF1	A1BG_BOVIN	A1BG	Alpha-1B-glycoprotein	100.0%	24.65	8	9
					CEAEVPDVSFLLLR	2	99.9%
					FPLGPVTSTTR	2	99.8%
					LEGEDQFLEVAEAPEATQATFPVHR	3	100.0%
					LSALAAGDGSGYTCR	2	100.0%
					SELAAWSR	2	97.7%
					SLLSELSDPVELR	2	100.0%
					VLRPGSSASLTCVAPLSGVDFQLR	3	100.0%
					VLSPAGPEAQFELR	2	100.0%
					VLSPAGPEAQFELR	3	100.0%
Q2UVX4	CO3_BOVIN	C3	Complement C3	100.0%	40.64	52	61
					AAVYNHFISDGVK	2	100.0%
					ACEPGVDYVYK	2	99.1%
					AGDFLENHYR	2	99.4%
					AGDFLENHYR	3	98.1%
					AGQYSSDLRK	2	100.0%
					AQFILQGDACVK	2	99.9%
					AYYEDSPQQVFSAEFEVK	2	100.0%
					DICEAQVNSLGR	2	99.9%
					DPLTITVR	2	99.3%
					DSITTWEILAVSLSDKK	2	97.0%
					EEVPAADLSDQVPDTESETK	2	100.0%
					EVTLEDRLDK	2	95.4%
					EVVADSVWVDVK	2	99.4%
					EYVLPSFEVQLEPEEK	2	99.9%
					FFKPAMPFDLMVYVTNPDGSPAR	2	99.2%
					FFKPAMPFDLMVYVTNPDGSPAR	3	100.0%
					FVTVVATFGNVQVEK	2	100.0%
					FYHPDKEDGMLSK	2	94.0%
					FYYIDDPDGLK	2	100.0%
					FYYIDDPDGLKVNIIAR	3	99.8%
					GQGTLSVVTVYHAK	2	93.7%
					GQGTLSVVTVYHAK	3	100.0%
					GSMILDICTK	2	99.1%
					GYTQQLAFR	2	99.2%
					HIPVVTQGSNVQSLTQDDGVAK	2	100.0%

					HIPVVTQGSNVQSLTQDDGVAK	3	100.0%
					HQQTITIPAR	2	100.0%
					IGLHEVEVK	2	95.4%
					ILLQGTPVAQMTEDAIDGER	2	99.7%
					ILLQGTPVAQMTEDAIDGER	3	99.8%
					ISLTHSLTR	2	98.0%
					KDYDTTPPVVR	2	100.0%
					KGYTQQLAFR	3	91.2%
					KQVLSNENTQLNSNNGYLSTVTIK	3	100.0%
					LPYSVVR	2	81.3%
					LSINTQNKR	2	99.0%
					NTLIIYLDK	2	84.6%
					NYAGVFTDAGLTLK	2	100.0%
					QVLLNGVQPSR	2	95.7%
					QVLSNENTQLNSNNGYLSTVTIK	2	100.0%
					QVLSNENTQLNSNNGYLSTVTIK	3	100.0%
					RQESLELIR	2	99.6%
					SDLDDDIIPEEDIISR	2	100.0%
					SSVAVPYVIVPLK	2	98.7%
					TGIPIVTSPYQIHFTK	2	99.6%
					TGIPIVTSPYQIHFTK	3	94.0%
					TIYTPGSTVLYR	2	98.1%
					TMQALPYNTQGNSNNYLHLSVPR	3	99.7%
					TSQGLETQQR	2	99.8%
					VELKPGETLNVNFHLR	3	100.0%
					VELLYNPAFCSLATAK	2	100.0%
					VHQYFNVGLIQPGAVK	3	92.8%
					VPINDGNGEAILK	2	100.0%
					VPINDGNGEAILKR	2	98.1%
					VPINDGNGEAILKR	3	100.0%
					VSHTVEDCLSFK	3	99.9%
					VSIRPAPETVK	2	99.6%
					VSIRPAPETVK	3	100.0%
					VYSYYNLDETCIR	2	100.0%
					YLGDQDATMSILDISMMTGFSPDVEDLK	2	100.0%
					YYGGGYGSTQATFMVFQALAQYQK	3	100.0%
Q3MHN5	VTDB_BOVIN	GC	Vitamin D-binding proteir	100.0%	29.54	10	11
					FMYEYSINYGQAPLTLLVGYTK	3	100.0%
					FPDATETDLQELVAK	2	100.0%
					GKFPDATETDLQELVAK	2	100.0%
					GKFPDATETDLQELVAK	3	100.0%
					GQELCADYSENTFTEYK	2	100.0%
					HQPQEFPTYVEPTNDEICEAFR	3	100.0%
					ICSQYAAYGK	2	99.9%
					KTQIPEVFLTK	2	96.8%
					LCDNLSTK	2	94.4%
					VLDQYIFELSR	2	100.0%
					VPTAHLEDVLPLAEDITTILSK	3	100.0%
Q3SYR0	Q3SYR0_BOVIN	SERPINA7	Serpin peptidase inhibitor, clade A (Alpha-1 antiprote	100.0%	18.25	5	5
					AQWANPFDPSKTEEGSSFLVDK	3	99.8%
					EGQMEWVEGAMSSK	2	100.0%
					MGIQDAFADNADFSGLTK	2	100.0%
					MSSINADFAFNLYR	2	100.0%
					VVDPTEV	1	88.2%
Q3SZ57	FETA_BOVIN	AFP	Alpha-fetoproteir	100.0%	53.93	24	34
					AAPASIPPFPVLEPVTSCK	2	99.9%
					AITVTK	1	96.6%
					APQLTSPELMALTR	2	100.0%
					DFNQLSSR	2	95.8%
					DLCQVQGVPLQTMK	2	100.0%
					DVLTIIEKPTGSK	2	100.0%
					DVLTIIEKPTGSK	3	92.3%
					ESSLLNQHICAVMGK	2	100.0%
					ESSLLNQHICAVMGK	3	95.2%
					FLGDRDFNQLSSR	2	97.9%
					FLGDRDFNQLSSR	3	93.5%
					GYQELLEK	2	99.9%
					HDCFLAHK	2	99.9%
					HEENPINPGVDQCCTSSYSNR	3	100.0%
					KAPQLTSPELMALTR	2	99.9%
					KAPQLTSPELMALTR	3	100.0%
					KMANAGAICCHLSEDK	3	99.9%
					LAVPIILR	2	99.7%
					LGEYYLQNAFLVAYTK	2	100.0%
					LGEYYLQNAFLVAYTK	3	100.0%
					LPTTLELGHCIIHAENDDKPEGLSPNVNR	3	100.0%
					QKPQITEEQLETVVADFSGLLEK	2	98.4%
					QKPQITEEQLETVVADFSGLLEK	3	100.0%
					QPAGCLENQVSAFLEEICR	2	100.0%
					QPAGCLENQVSAFLEEICR	3	100.0%
					QQFLINLVK	2	96.8%
					RHPVLYAPTILSVANQYNK	2	100.0%
					RHPVLYAPTILSVANQYNK	3	100.0%
					RPCFSSLVVDETYVPPPFSDDKFIFHK	3	98.1%
					SCGLFQK	1	81.9%
					SCGLFQK	2	92.3%
					VMSYICSQQDILSR	2	100.0%
					YIQESQALAK	1	100.0%
					YIQESQALAK	2	99.8%
Q3SZH5	Q3SZH5_BOVIN	AGT	Angiotensinogen (Serpin peptidase inhibitor, clade A	100.0%	18.63	4	4
					FMQSVTGWNMGR	2	99.8%
					QPFVQSLSSFAPITLPR	2	100.0%
					SNCDQLEKPSVETPPDPTFTPVPIQTK	3	100.0%
					VLSSLQTIQGLLVAQGGASSQAR	3	100.0%
Q3SZR3	A1AG_BOVIN	ORM1	Alpha-1-acid glycoproteir	100.0%	52.97	13	17
Q5GN72	Q5GN72_BOVIN	agp	Alpha-1 acid glycoprotein (Precursor)	100.0%	52.97	13	17
					AIQAAFFYLEPR	2	100.0%
					CIGIQESEIIYTDEK	2	100.0%
					CIGIQESEIIYTDEKK	2	100.0%
					CIGIQESEIIYTDEKK	3	100.0%

					EFLDVIKCIGIQESEIIYTDEK	3	93.2%
					EHFVDLLLSK	2	99.8%
					EYQTIEDK	1	98.7%
					EYQTIEDK	2	83.0%
					HAEDKLITR	2	99.9%
					KEFLDVIK	2	97.4%
					NVGVSFYADKPEVTQEQK	2	100.0%
					NVGVSFYADKPEVTQEQK	3	100.0%
					NVGVSFYADKPEVTQEQKK	2	100.0%
					NVGVSFYADKPEVTQEQKK	3	100.0%
					TFMLAASWNGTK	2	100.0%
					VESDREHFVDLLLSK	3	100.0%
					WFYIGSAFR	2	100.0%
Q3SZV7	HEMO_BOVIN	HPX	Hemopexin	100.0%	31.15	10	12
					AGYTLVK	2	97.3%
					APELGHGVEGGNVAKPDPEVTER	3	100.0%
					CSDGWGFDATTLDEHGNMLFLK	3	97.1%
					DLPQAQR	2	90.0%
					DYFMSCPNR	2	100.0%
					IEHLWPQGPSTVDAAFLWDK	3	100.0%
					LDLNLGAQATWTELPWLHTK	2	91.5%
					LDLNLGAQATWTELPWLHTK	3	100.0%
					MNSLLGCAPHQHS	3	92.6%
					VDGALCTEK	2	98.4%
					WKDAPSPVDAAFR	2	97.9%
					WKDAPSPVDAAFR	3	100.0%
Q3Y5Z3	ADIPO_BOVIN	ADIPOQ	Adiponectin	98.0%	11.25	2	2
					ALLFTHDQFQDK	2	98.0%
					NVDQASGSVLLYLEK	2	97.6%
Q3ZBS7	Q3ZBS7_BOVIN	VTN	Uncharacterized protein	100.0%	13.87	5	5
					DSWVDIFR	2	98.2%
					DVWGIEGPIDAAFTR	2	100.0%
					FQDGVLEPDFPR	2	100.0%
					LDAAMAGHIYVSGSAPSFPR	3	100.0%
					RVDAVIPPYPR	2	99.8%
Q58CQ9	VNN1_BOVIN	VNN1	Pantetheinase	100.0%	10.78	4	4
					DSAPNTLSDLTTQALR	2	100.0%
					MTGSGIYAPDSPR	2	100.0%
					NLDLLEGAVTSASK	2	100.0%
					YQYNTDVVFDSK	2	100.0%
Q58D62	FETUB_BOVIN	FETUB	Fetuin-B	100.0%	32.56	11	14
					AIFYINK	2	98.8%
					DGYVLSLNR	2	99.9%
					FMETATESLAK	1	98.7%
					FMETATESLAK	2	100.0%
					GSVQYLPDWDK	2	99.6%
					GSVQYLPDWDKK	3	100.0%
					GYPFILPS	1	94.4%
					GYPFILPS	2	95.6%
					IFHESVYGQCK	3	99.7%
					IISVTCSFFNSQAPTPR	2	100.0%
					KDGYVLSLNR	2	100.0%
					SEGSSCALESPGSVPVGICHGSLGEPQGNQGK	3	100.0%
					TSSQWVFGPAYFVEYLIK	2	100.0%
					TSSQWVFGPAYFVEYLIK	3	100.0%
Q5BIR6	MED17_BOVIN	MED17	Mediator of RNA polymerase II transcription subunit 1	99.9%	5.84	2	2
					FMTLDPVSQDALPPK	3	99.9%
					NNLRSALTEMCVLYDVLSIVRDK	3	99.9%
Q7SIH1	A2MG_BOVIN	A2M	Alpha-2-macroglobulin	100.0%	48.08	53	67
					AAQVTIQSSGTFSTK	2	100.0%
					AILYLNTGYQR	2	100.0%
					ALLAYAFALAGNQER	2	100.0%
					ALLAYAFALAGNQER	3	100.0%
					ASFSVLGDILGSAMR	2	100.0%
					ASFSVLGDILGSAMR	3	100.0%
					ATVLNYLPK	2	99.9%
					DFVHFDDTSEPPTETVR	2	100.0%
					DFVHFDDTSEPPTETVR	3	99.8%
					DMYSFLQDMGLK	2	100.0%
					DTGLGLSPTASLR	2	100.0%
					DTIIKPLLVEPEGLEK	3	100.0%
					FEVQVR	1	95.5%
					FQVENSNR	1	97.9%
					FSGELNNEGCFSQQVNTK	2	100.0%
					GATEITTTITK	2	99.6%
					GGVDDEVTLSAYITIALLEMPLPVTHPVVR	2	99.2%
					GNSEGNTWLTAFVLK	2	100.0%
					GSGGTAEHPFTVEEFVLPK	2	100.0%
					GSGGTAEHPFTVEEFVLPK	3	97.6%
					GVPMPNK	1	95.2%
					GYIFIDEAHITEALTWLAQK	2	100.0%
					GYIFIDEAHITEALTWLAQK	3	100.0%
					HFDGSYSTFGEHR	3	100.0%
					HFPPAAATDTAADAHDPARPGAK	3	99.9%
					IAQWQNLEVENGLQQLTFPLSSEPFQGSYK	2	100.0%
					IAQWQNLEVENGLQQLTFPLSSEPFQGSYK	3	100.0%
					IQEEGTEVELTGK	2	100.0%
					IQHHTLLASPVR	3	100.0%
					KDEFPFALEVQTLPQTCDGPK	3	99.9%
					KDTIIKPLLVEPEGLEK	3	100.0%
					LLIYAILPDGEVVGDSAR	2	100.0%
					LLIYAILPDGEVVGDSAR	3	99.8%
					LLTEDIYQPR	2	100.0%
					LPPNVVEESAR	2	100.0%
					LSFVTVDSNLR	2	100.0%
					LSFVTVDSNLRR	3	82.5%
					MVSGFIPLKPTVK	2	100.0%
					MVSGFIPLKPTVK	3	99.8%
					NALFCLDSAWK	2	99.4%
					NEESLVFVQTDKPIYKPEQTVK	3	100.0%

					NPSNCYGEESNAVCEK	2	100.0%
					NRDFVHFDDTSEPPTETVR	3	100.0%
					NRDFVHFDDTSEPPTETVRK	3	99.9%
					PASNMAIVDVK	2	100.0%
					PQYMVLVPSLLHTETPEK	3	97.8%
					QQNCQGGFSSTQDTVVALHALSR	3	100.0%
					QTVSWAVTPK	2	99.1%
					RQEFEMK	2	97.6%
					SLFTDVVAEK	2	99.8%
					SNSFVYLEPLPR	2	100.0%
					SNSFVYLEPLPR	3	99.2%
					SPTSQEVMFLTIQVK	2	100.0%
					SPTSQEVMFLTIQVK	3	100.0%
					STGTLLNNAIK	2	99.2%
					TEVSNNHVLIYLDK	2	100.0%
					TEVSNNHVLIYLDK	3	99.3%
					TQTVQAHYVLK	2	100.0%
					TQTVQAHYVLK	3	99.7%
					VGLNFSPGQSFPASQAHLR	2	100.0%
					VGLNFSPGQSFPASQAHLR	3	100.0%
					VSVQLEASPAFLAVPEK	2	99.9%
					VTASPQSLCALR	2	100.0%
					VTNETLTLTFTVLQDIPVR	2	100.0%
					VYDYYETDEFAVAEYSAPCSK	2	100.0%
					VYDYYETDEFAVAEYSAPCSK	3	90.6%
					YGAATFTSAR	2	99.8%
Q95121	PEDF_BOVIN	SERPINF1	Pigment epithelium-derived factor	100.0%	39.42	11	13
					ALYYDLISNPDIHGTYK	3	100.0%
					IAQLPLTGSTSIIFFLPQK	2	100.0%
					KTSLEDFYLDEER	2	98.7%
					KTSLEDFYLDEER	3	99.4%
					LAAAVSNFGYDLYR	2	100.0%
					LQSLFDAPDFSK	2	99.8%
					SGESPTANVLLSPLSVATALSALSLGAEQR	2	100.0%
					SGESPTANVLLSPLSVATALSALSLGAEQR	3	100.0%
					TSLEDFYLDEER	2	99.9%
					VDLQEINNWVQAQMK	2	100.0%
					VGFEWNEDGAGTNSSPGVQPAR	2	100.0%
					VPMMSDPQAVLR	2	99.9%
					YGLDSDLNCK	2	100.0%
Q9N2I2	IPSP_BOVIN	SERPINA5	Plasma serine protease inhibitor	100.0%	8.66	3	3
					DFVFDLYR	2	96.5%
					FSIEGSYQLEEVLPK	2	100.0%
					LPIQEAFLGAMR	2	100.0%
Q9TT36	THBG_BOVIN	SERPINA7	Thyroxine-binding globulin	100.0%	22.63	6	6
					AQWANPFDPSKTEEGSSFLVDK	3	99.8%
					EGQMEWVEGAMSSK	2	100.0%
					FLNQPETTLLHPIIQFDR	3	97.4%
					MGIQDAFADNADFSGLTK	2	100.0%
					MSSINADFAFNLYR	2	100.0%
					VVDPTEA	1	97.3%
Q9TTE1	SPA31_BOVIN	SERPINA3-1	Serpin A3-1	100.0%	42.34	15	20
					DTQSIIFLGK	2	99.9%
					FIEDAQVLYSSEAFPTNFR	2	100.0%
					FIEDAQVLYSSEAFPTNFR	3	100.0%
					GSTLTEILEGLK	2	100.0%
					LLDKFIEDAQVLYSSEAFPTNFR	2	100.0%
					LLDKFIEDAQVLYSSEAFPTNFR	3	100.0%
					NPNKNVILSPLSVSIALAFLSLGAR	3	99.5%
					RIHELYLPK	2	99.8%
					RIHELYLPK	3	100.0%
					RVDGHTLASSNTDFAFSLYK	3	100.0%
					SLINDYVK	2	94.6%
					SNYELNDILSQLGIR	2	100.0%
					SNYELNDILSQLGIR	3	100.0%
					SNYELNDILSQLGIRK	3	88.4%
					TELVLVNYIYFK	2	100.0%
					TQGKIEELFK	2	86.4%
					TVEVPMMTLDLETPYFR	2	100.0%
					VDGHTLASSNTDFAFSLYK	2	100.0%
					VDGHTLASSNTDFAFSLYK	3	100.0%
					VNRPFLIAIVLK	2	97.5%
