# Supplementary material for: Silver Nanoparticle Protein Corona Composition in Cell Culture Media
Source: PLoS One. 2013 Sep 9;8(9):e74001. doi: 10.1371/journal.pone.0074001 (PMC3767594; doi:10.1371/journal.pone.0074001)
Supplement: Table S2 — Quantification of all proteins and peptides found to associate in the corona of each AgNP by label-free mass spectroscopy. (PDF) [file pone.0074001.s002.pdf]

Protein ID	Protein Entry	Gene Name	Protein Name	mean 20nm Citrate	mean 20nm PVP	mean 110nm Citrate	mean 110nm PVP	SEM 20nm Citrate	SEM 20nm PVP	SEM 100nm Citrate	SEM 100nm PVP
E1B7E5	E1B7E5_BOVIN	RPS12	40S ribosomal protein S12	13,942,181	14,064,267	-	-	367,887	1,185,072	-	-
F1MH27	F1MH27_BOVIN	CHIA	Acidic mammalian chitinase	-	-	-	4,515,283	-	-	-	97,258
P60712	ACTB_BOVIN	ACTB	Actin, cytoplasmic 1	-	-	708,492	1,839,823	-	-	71,095	300,901
F1MRD0	F1MRD0_BOVIN	ACTG1	Actin, cytoplasmic 2	-	-	599,584	1,016,290	-	-	36,520	53,773
E1BK41	E1BK41_BOVIN	ADAMTSL1	ADAMTS-like 1	-	557,764	-	-	-	44,176	-	-
Q3Y5Z3	ADIPO_BOVIN	ADIPOQ	Adiponectin	-	-	-	5,162,717	-	-	-	428,408
Q3SZR3	A1AG_BOVIN	ORM1	Alpha-1-acid glycoprotein	-	-	20,111,109	43,586,334	-	-	2,538,916	5,493,987
P34955	A1AT_BOVIN	SERPINA1	Alpha-1-antiproteinase	137,751,264	196,573,274	1,107,447,117	1,714,877,708	5,117,698	9,316,801	90,297,612	45,754,707
Q2KJF1	A1BG_BOVIN	A1BG	Alpha-1B-glycoprotein	-	-	13,665,289	39,563,448	-	-	3,237,439	2,551,620
F1MJK3	F1MJK3_BOVIN	A1M	Alpha-1-macroglobulin	-	-	10,938,963	24,316,893	-	-	972,588	462,391
P28800	A2AP_BOVIN	SERPINF2	Alpha-2-antiplasmin	-	-	112,548,165	127,813,382	-	-	4,232,940	5,727,231
P12763	FETUA_BOVIN	AHSG	Alpha-2-HS-glycoprotein	42,987,189	55,358,017	1,051,623,127	1,860,807,355	943,763	3,305,993	107,945,722	66,349,576
Q7SIH1	A2MG_BOVIN	A2M	Alpha-2-macroglobulin	-	-	160,172,589	351,314,570	-	-	20,128,120	32,426,349
Q3ZC55	ACTN2_BOVIN	ACTN2	Alpha-actinin-2	1,209,088	1,138,938	-	-	50,252	121,816	-	-
Q3SZ57	FETA_BOVIN	AFP	Alpha-fetoprotein	-	-	139,157,989	298,562,931	-	-	17,351,876	29,217,525
O18920	ANGP1_BOVIN	ANGPT1	Angiopoietin-1	-	1,312,423	10,592,624	-	-	120,896	1,258,212	-
Q3SZH5	Q3SZH5_BOVIN	AGT	Angiotensinogen	-	-	826,275	4,055,386	-	-	184,417	347,708
F1MZN6	F1MZN6_BOVIN	ANGT	Angiotensinogen	-	-	10,375,355	42,122,191	-	-	2,074,147	5,550,611
P41361	ANT3_BOVIN	SERPINC1	Antithrombin-III	-	-	10,487,213	26,212,116	-	-	1,504,322	3,263,772
E1BMW2	E1BMW2_BOVIN	AP2A1	AP-2 complex subunit alpha-1	-	1,701,020	-	-	-	274,783	-	-
P15497	APOA1_BOVIN	APOA1	Apolipoprotein A-I	53,092,606	67,806,812	629,799,623	926,742,414	2,844,689	2,909,308	82,827,428	25,461,687
P81644	APOA2_BOVIN	APOA2	Apolipoprotein A-II	6,540,054	4,736,466	7,663,645	32,183,017	393,026	547,091	1,757,584	5,229,521
Q32PJ2	APOA4_BOVIN	APOA4	Apolipoprotein A-IV	-	-	5,961,029	5,839,625	-	-	1,510,554	570,760
E1BNR0	E1BNR0_BOVIN	APOB	Apolipoprotein B-100	-	76,988,867	133,301,211	230,246,258	-	1,866,976	13,212,783	28,905,104
P19035	APOC3_BOVIN	APOC3	Apolipoprotein C-III	1,756,191	1,573,732	6,085,616	8,698,216	69,851	115,347	670,253	681,628
Q03247	APOE_BOVIN	APOE	Apolipoprotein E	-	-	5,061,038	7,359,267	-	-	374,041	203,387
P17690	APOH_BOVIN	APOH	Beta-2-glycoprotein 1	-	-	66,478,290	79,478,167	-	-	4,971,221	1,570,860
F1N2G1	F1N2G1_BOVIN	BOD1L	BOD1L protein	5,285,700	3,852,633	-	-	116,642	269,957	-	-
P35445	COMP_BOVIN	COMP	Cartilage oligomeric matrix protein	-	-	1,107,876	2,132,266	-	-	234,906	231,791
P17697	CLUS_BOVIN	CLU	Clusterin	-	-	6,152,038	13,619,176	-	-	894,453	629,244
F1MEZ4	F1MEZ4_BOVIN	CCDC82	Coiled-coil domain containing 82	-	4,096,417	-	-	-	67,815	-	-
P02453	CO1A1_BOVIN	COL1A1	Collagen alpha-1(I) chain	1,006,708	2,740,198	-	-	135,305	444,277	-	-
E1BI98	E1BI98_BOVIN	COL6A1	Collagen alpha-1(VI) chain	-	-	3,223,863	6,664,167	-	-	533,620	697,980
Q2UVX4	CO3_BOVIN	C3	Complement C3	36,823,758	-	95,498,520	169,057,990	159,306	-	10,634,513	9,078,718
P01030	CO4_BOVIN	C4	Complement C4	-	-	26,224,494	43,178,667	-	-	1,454,820	3,311,710
F1MY85	F1MY85_BOVIN	C5	Complement C5	-	-	-	329,060	-	-	-	38,633
P81187	CFAB_BOVIN	CFB	Complement factor B	-	-	28,036,165	49,182,392	-	-	2,305,696	2,736,573
Q28085	CFAH_BOVIN	CFH	Complement factor H	-	-	11,475,168	17,418,908	-	-	2,141,752	2,135,769
F1N4M7	F1N4M7_BOVIN	CF1	Complement factor I	-	-	4,153,252	11,525,359	-	-	564,483	859,238
F1N290	F1N290_BOVIN	COBLL1	Cordon-bleu protein-like 1	1,069,578	1,046,805	-	-	21,695	134,481	-	-
E1BF81	E1BF81_BOVIN	SERPINA6	Corticosteroid-binding globulin	-	-	848,949	2,590,591	-	-	130,266	605,609
B8Y9T0	B8Y9T0_BOVIN	FN1	Cumulus cell-specific fibronectin 1	259,964	-	1,073,722	-	13,890	-	236,389	-
P01035	CYTC_BOVIN	CST3	Cystatin-C	-	-	-	1,030,016	-	-	-	248,117
F1MPT5	F1MPT5_BOVIN	DST	Dystonin	3,511,638	4,037,217	-	-	386,033	630,825	-	-
Q58D62	FETUB_BOVIN	FETUB	Fetuin-B	-	-	65,198,870	113,069,729	-	-	7,165,537	5,817,475
A5PJE3	A5PJE3_BOVIN	FGA	Fibrinogen alpha chain	-	-	1,016,464	3,452,314	-	-	220,414	184,798
F1MYN5	F1MYN5_BOVIN	FBLN1	Fibulin-1	-	-	8,563,732	15,000,959	-	-	977,632	249,686
F1N1I6	F1N1I6_BOVIN	GSN	Gelsolin	-	-	5,891,517	12,016,943	-	-	674,063	1,190,771
E1BK20	E1BK20_BOVIN	GCKR	Glucokinase regulatory protein	-	2,656,407	-	-	-	218,253	-	-
F1MLZ9	F1MLZ9_BOVIN	GRIN2A	Glutamate [NMDA] receptor subunit epsilon-1	2,839,052	-	-	-	545,631	-	-	-
P02081	HBBF_BOVIN	HBBF	Hemoglobin fetal subunit beta	-	-	47,312,509	76,951,809	-	-	6,792,620	6,929,504
P01966	HBA_BOVIN	HBA	Hemoglobin subunit alpha	-	-	29,322,527	55,438,087	-	-	3,241,787	4,036,912
P02070	HBB_BOVIN	HBB	Hemoglobin subunit beta	-	-	1,089,320	5,117,297	-	-	323,393	896,434
Q3SZV7	HEMO_BOVIN	HPX	Hemopexin	-	-	26,749,606	35,642,403	-	-	524,155	2,503,278

A6QPP2	A6QPP2_BOVIN	SERPIND1	Heparin cofactor 2	-	-	3,986,561	21,648,489	-	-	559,993	1,217,496
E1BCW0	E1BCW0_BOVIN	HGFAC	Hepatocyte growth factor activator	-	-	-	5,656,261	-	-	-	816,264
F1MYZ3	F1MYZ3_BOVIN	MLL3	Histone-lysine N-methyltransferase	242,545,733	-	-	-	8,100,462	-	-	-
G5E5T5	G5E5T5_BOVIN	IGHM	Ig mu chain C	-	-	-	209,449	-	-	-	45,147
F1MLW7	F1MLW7_BOVIN	IGL@	Immunoglobulin light chain, lambda gene cluster	-	-	2,000,183	5,767,706	-	-	98,781	634,167
Q0VCM5	ITIH1_BOVIN	ITIH1	Inter-alpha-trypsin inhibitor heavy chain H1	-	-	48,215,394	127,244,285	-	-	13,575,474	23,811,461
F1MNW4	F1MNW4_BOVIN	ITIH2	Inter-alpha-trypsin inhibitor heavy chain H2	-	-	42,749,950	76,263,813	-	-	4,024,389	5,302,752
P56652	ITIH3_BOVIN	ITIH3	Inter-alpha-trypsin inhibitor heavy chain H3	-	-	46,297,661	76,550,606	-	-	6,887,817	3,226,999
Q3T052	ITIH4_BOVIN	ITIH4	Inter-alpha-trypsin inhibitor heavy chain H4	-	-	21,355,682	44,043,887	-	-	1,032,786	2,084,240
F1MXJ6	F1MXJ6_BOVIN	KALRN	Kalirin	-	10,814,400	-	-	-	67,423	-	-
A6QNZ7	A6QNZ7_BOVIN	KRT10	Keratin, type I cytoskeletal 10	1,311,358	1,819,177	5,338,630	10,235,567	71,854	112,441	1,681,943	1,080,164
F1MC11	F1MC11_BOVIN	KRT13	Keratin, type I cytoskeletal 13	414,800	321,956	-	-	46,270	27,943	-	-
Q17QL7	Q17QL7_BOVIN	KRT15	Keratin, type I cytoskeletal 15	982,771	1,306,959	2,680,392	3,647,633	41,376	39,465	309,013	143,469
A1L595	K1C17_BOVIN	KRT17	Keratin, type I cytoskeletal 17	-	2,022,711	-	-	-	219,493	-	-
G3N0V2	G3N0V2_BOVIN	KRT1	Keratin, type II cytoskeletal 1	7,942,835	8,862,485	15,221,562	24,193,041	905,244	477,702	1,264,075	1,756,216
F1MGQ6	F1MGQ6_BOVIN	KRT6A	Keratin, type II cytoskeletal 6A	1,009,977	942,635	1,693,624	-	52,985	38,202	181,683	-
F1MUY2	F1MUY2_BOVIN	KRT6C	Keratin, type II cytoskeletal 6C	-	-	1,889,425	-	-	-	58,246	-
Q29S21	K2C7_BOVIN	KRT7	Keratin, type II cytoskeletal 7	558,937	652,257	102,519	467,117	6,830	22,923	20,074	55,154
G3MXL3	G3MXL3_BOVIN	KRT79	Keratin, type II cytoskeletal 79	1,324,620	1,340,164	466,830	1,437,697	143,244	28,650	70,542	152,568
P01044	KNG1_BOVIN	KNG1	Kininogen-1	-	-	23,273,813	27,329,584	-	-	727,446	2,808,617
P01045	KNG2_BOVIN	KNG2	Kininogen-2	-	-	19,115,470	26,334,363	-	-	1,860,799	2,039,210
A1A4K4	A1A4K4_BOVIN	LLGL1	Lethal giant larvae homolog 1 (Drosophila)	-	945,511	-	-	-	32,548	-	-
Q2KIF2	Q2KIF2_BOVIN	LRG1	Leucine-rich alpha-2-glycoprotein 1	-	-	4,793,781	18,350,770	-	-	578,834	2,992,833
E1BGJ0	E1BGJ0_BOVIN	LRP1	Low-density lipoprotein receptor-related protein 1 intracellular domair	7,582,318	7,684,025	-	-	1,586,725	325,662	-	-
E1BN03	E1BN03_BOVIN	LRP12	Low-density lipoprotein receptor-related protein 12	5,816,234	-	-	-	114,771	-	-	-
Q05443	LUM_BOVIN	LUM	Lumican	-	-	3,035,094	10,138,532	-	-	851,914	1,239,686
P43481	KIT_BOVIN	KIT	Mast/stem cell growth factor receptor Kit	3,611,762	3,096,026	-	-	303,492	316,212	-	-
Q5BIR6	MED17_BOVIN	MED17	Mediator of RNA polymerase II transcription subunit 17	2,057,492	1,754,802	-	-	264,633	152,780	-	-
G3MWW2	G3MWW2_BOVIN	TET1	Methylcytosine dioxygenase	-	3,121,311	-	-	-	70,833	-	-
E1BC24	E1BC24_BOVIN	MDN1	Midasin	4,876,754	-	-	-	361,726	-	-	-
Q58CQ9	VNN1_BOVIN	VNN1	Pantetheinase	-	-	4,513,474	9,907,800	-	-	585,076	532,915
G5E5D5	G5E5D5_BOVIN	PAXIP1	PAX-interacting protein 1	-	2,243,775	-	-	-	190,641	-	-
F1MT13	F1MT13_BOVIN	PDZD2	PDZ domain-containing protein 2	127,462,797	-	-	-	5,316,195	-	-	-
Q29RZ2	PPWD1_BOVIN	PPWD1	Peptidylprolyl isomerase domain and WD repeat-containing protein 1	-	14,653,683	-	-	-	1,319,887	-	-
E1BKZ0	E1BKZ0_BOVIN	PCNT	Pericentrin	13,363,362	-	-	-	1,210,508	-	-	-
Q95121	PEDF_BOVIN	SERPINF1	Pigment epithelium-derived factor	-	-	7,774,055	28,908,833	-	-	1,217,669	4,405,153
E1BMJ0	E1BMJ0_BOVIN	SERPING1	Plasma protease C1 inhibitor	-	-	5,033,426	17,081,979	-	-	926,777	2,196,412
Q9N2I2	IPSP_BOVIN	SERPINA5	Plasma serine protease inhibitor	-	-	719,140	3,760,242	-	-	158,671	486,779
P06868	PLMN_BOVIN	PLG	Plasminogen	-	-	7,621,493	10,625,672	-	-	127,729	983,624
P18493	PARP1_BOVIN	PARP1	Poly [ADP-ribose] polymerase 1	2,775,358	4,748,992	-	-	212,844	144,515	-	-
F1MMK9	F1MMK9_BOVIN	AMBP	Protein AMBP	-	-	4,445,300	9,624,090	-	-	497,045	695,345
Q2KIU3	HP252_BOVIN	HP-25	Protein HP-25 homolog 2	-	-	-	7,221,263	-	-	-	2,030,299
E1BNU3	E1BNU3_BOVIN	KANK3	Protein Kank3 (ankyrin repeat domain)	2,241,148	1,727,872	-	-	165,802	72,870	-	-
F1MPT4	F1MPT4_BOVIN	SDK2	Protein sidekick-2	-	-	-	13,362,160	-	-	-	1,402,449
E1BLI8	E1BLI8_BOVIN	SOGA1	Protein SOGA1	-	6,416,587	-	-	-	321,225	-	-
A5PJ69	A5PJ69_BOVIN	SERPINA10	Protein Z-dependent protease inhibitor	-	-	-	474,504	-	-	-	218,240
P00735	THRB_BOVIN	F2	Prothrombin	-	-	8,710,954	23,459,729	-	-	2,493,010	3,643,362
P18902	RET4_BOVIN	RBP4	Retinol-binding protein 4	-	-	1,230,241	1,838,839	-	-	210,506	130,972
F1MMG6	F1MMG6_BOVIN	SCARA3	Scavenger receptor class A member 3	-	3,066,624	-	-	-	166,137	-	-
E1BA03	E1BA03_BOVIN	PAK6	Serine/threonine-protein kinase	2,153,643	2,151,897	-	-	213,650	266,513	-	-
Q29443	TRFE_BOVIN	TF	Serotransferrin	-	-	270,081,329	454,277,067	-	-	29,425,839	21,127,340
Q9TTE1	SPA31_BOVIN	SERPINA3-1	Serpin A3-1	-	-	12,559,276	35,330,572	-	-	2,311,114	6,109,768
A2I7M9	SPA32_BOVIN	SERPINA3-2	Serpin A3-2	-	-	12,290,560	33,355,107	-	-	2,219,734	5,470,508
Q3ZEJ6	SPA33_BOVIN	SERPINA3-3	Serpin A3-3	-	-	289,204	1,499,083	-	-	76,208	304,862
G3N1U4	G3N1U4_BOVIN	SPA33	Serpin A3-3	-	-	9,549,611	19,987,503	-	-	1,482,074	1,394,298
E1B8H1	E1B8H1_BOVIN	SERPINA3-6	Serpin A3-6	-	-	26,397,331	42,518,893	-	-	491,476	3,133,122

F1MXV8	F1MXV8_BOVIN	SERPINA3-7	Serpin A3-7	-	-	10,509,328	23,859,902	-	-	1,317,794	1,477,375
P02769	ALBU_BOVIN	ALB	Serum albumin	142,328,886	140,408,089	3,581,910,422	7,673,438,622	2,033,770	7,636,946	565,759,667	720,864,333
E1BGL8	E1BGL8_BOVIN	SIGLEC1	Sialoadhesin	1,342,191	2,007,710	-	-	85,794	151,816	-	-
E1BCK9	E1BCK9_BOVIN	SNAPC4	snRNA-activating protein complex subunit 4	919,543	646,642	-	-	129,686	76,016	-	-
A0JN83	A0JN83_BOVIN	SLC25A44	Solute carrier family 25, member 44	-	6,780,850	-	-	-	401,729	-	-
F1MKE9	F1MKE9_BOVIN	SPTB	Spectrin beta chain	8,543,233	7,768,597	-	-	65,702	368,600	-	-
G3N022	G3N022_BOVIN	SPEG	Striated muscle preferentially-expressed protein kinase	4,513,921	-	-	-	505,379	-	-	-
F6RF21	F6RF21_BOVIN	SMCHD1	Structural maintenance of chromosomes flexible hinge domain-contair	-	7,334,983	-	-	-	266,187	-	-
Q2KIS7	TETN_BOVIN	CLEC3B	Tetranectin	-	-	515,727	2,601,065	-	-	117,202	315,998
Q28178	TSP1_BOVIN	THBS1	Thrombospondin-1	20,445,705	-	20,156,295	38,892,391	290,321	-	1,705,695	560,604
Q9TT36	THBG_BOVIN	SERPINA7	Thyroxine-binding globulin	-	-	1,816,200	5,690,362	-	-	351,222	1,301,966
F1N2D3	F1N2D3_BOVIN	ZO1	Tight junction protein ZO-1	14,518,250	-	-	-	1,428,856	-	-	-
F1N757	F1N757_BOVIN	TTN	Titin	15,264,211	8,383,767	-	-	697,580	810,482	-	-
E1BEB8	E1BEB8_BOVIN	SUPT6H	Transcription elongation factor SPT6	3,836,410	4,110,691	-	-	169,428	159,643	-	-
O46375	TTHY_BOVIN	TTR	Transthyretin	-	-	25,016,623	71,778,777	-	-	2,534,089	12,472,137
Q3MHN5	VTDB_BOVIN	GC	Vitamin D-binding protein	-	-	11,041,729	36,193,503	-	-	1,619,200	5,217,519
F1N5M2	F1N5M2_BOVIN	VTDB	Vitamin D-binding protein	-	-	24,106,551	73,827,357	-	-	4,072,402	6,555,810
P07224	PROS_BOVIN	PROS1	Vitamin K-dependent protein S	-	-	-	5,426,924	-	-	-	636,394
Q3ZBS7	Q3ZBS7_BOVIN	VTN	Vitronectin	7,838,985	-	4,897,482	22,334,333	211,924	-	1,177,550	3,435,962
E1B9V7	E1B9V7_BOVIN	WDR37	WD repeat-containing protein 37	4,115,664	-	-	-	121,213	-	-	-
G3N1S7	G3N1S7_BOVIN	WDR52	WD repeat-containing protein 52	-	2,758,260	-	-	-	256,702	-	-
G3N2D0	G3N2D0_BOVIN	ZNF469	Zinc finger protein 469	13,579,517	-	-	-	695,234	-	-	-
E1BJV5	E1BJV5_BOVIN	ZNF638	Zinc finger protein 638	4,109,583	3,505,628	-	-	928,760	234,542	-	-
